# Effects of Sugars and Sugar Alcohols on the Gelatinization Temperatures of Wheat, Potato, and Corn Starches

**DOI:** 10.3390/foods9060757

**Published:** 2020-06-08

**Authors:** Matthew C. Allan, MaryClaire Chamberlain, Lisa J. Mauer

**Affiliations:** Department of Food Science, Purdue University, 745 Agriculture Mall Drive, West Lafayette, IN 47907, USA; wapatomatt@gmail.com (M.C.A.); mnchambe@ncsu.edu (M.C.)

**Keywords:** sugar, sugar alcohols, sweeteners, gelatinization, starch

## Abstract

The gelatinization temperature (T_gel_) of starch increases in the presence of sweeteners due to sweetener-starch intermolecular interactions in the amorphous regions of starch. Different starch botanical sources contain different starch architectures, which may alter sweetener-starch interactions and the effects of sweeteners on T_gel_s. To document these effects, the T_gel_s of wheat, potato, waxy corn, dent corn, and 50% and 70% high amylose corn starches were determined in the presence of eleven different sweeteners and varying sweetener concentrations. T_gel_s of 2:1 sweetener solution:starch slurries were measured using differential scanning calorimetry. The extent of T_gel_ elevation was affected by both starch and sweetener type. T_gel_s of wheat and dent corn starches increased the most, while T_gel_s of high amylose corn starches were the least affected. Fructose increased T_gel_s the least, and isomalt and isomaltulose increased T_gel_s the most. Overall, starch T_gel_s increased more with increasing sweetener concentration, molar volume, molecular weight, and number of equatorial and exocyclic hydroxyl groups. Starches containing more short amylopectin chains, fewer amylopectin chains that span through multiple clusters, higher number of building blocks per cluster, and shorter inter-block chain lengths exhibited the largest T_gel_ increases in sweetener solutions, attributed to less stable crystalline regions.

## 1. Introduction

Starch granules are botanical energy storage vessels that differ in size (2–100 μm) and molecular architecture between botanical sources, although all native starches share the traits of being semicrystalline, water insoluble, and composed predominantly of two α-glucans: amylopectin and amylose [1,2,3]. Amylose is primarily a linear polysaccharide with a few branch points and a degree of polymerization (DP) ranging from 600 to 6000 [3]. Amylose resides in the amorphous regions of the starch granule, especially near the periphery, although some amylose may also be co-crystallized with amylopectin [2]. Amylopectin is a larger, branched molecule (5% branching) that has a DP of 9600 to >15,900 [3]. The regions near amylopectin branches are amorphous, while the unbranched regions of amylopectin form crystalline, double helical structures [4]. These alternating amorphous and crystalline lamellae have a repeating distance of ~9 nm, regardless of the botanical source [5]. On a larger scale, starch granules also have alternating hard crystalline and softer semicrystalline growth rings (also known as shells) that are 100 to 400 nm thick [4]. Within these growth rings are blocklets that range in diameter from 20–500 nm, with smaller blocklets in the semicrystalline growth rings and larger blocklets in the crystalline growth rings. In general, A-type crystalline starches (e.g., cereals) have smaller blocklets than B-type crystalline starches (e.g., tubers) [4,6]. The amorphous lamella and spaces between blocklets result in the semicrystalline starch structure (15–42% crystalline). This allows for water to passively diffuse into the starch granule (up to 35% moisture content wet basis in starch) and cause reversible swelling and plasticization [2,7]. With the application of sufficient thermal energy (>57.1 to 72.0 °C onset temperature range reported in Ratnayake and Jackson [8]), the molecular mobility in the amorphous regions is great enough to strip apart and melt the crystalline double helices in a process known as gelatinization [9,10]. The temperature at which this occurs is the gelatinization temperature (T_gel_). 

Despite the commonality that starch gelatinization is the melting of amylopectin crystals, the T_gel_s of starches from different botanical sources vary [11]. Structural factors that influence the T_gel_s of starches include: the crystalline form of starch [12], amount of crystal defects [13], amylopectin structure [11,14,15], and amylose content [16,17]. Branch lengths of amylopectin have been extensively correlated with the T_gel_s of starches: starches with higher amounts of short amylopectin chains (DP 6–12) have lower T_gel_s, and starches with higher percentages of longer amylopectin chains (DP 14–25) have higher T_gel_s [18,19,20]. Additionally, starches with fewer building blocks (densely branched regions of amylopectin that precede double helices) per cluster (group of amylopectin branches in close proximity, NBbl) and greater spacing between these building blocks (IB-CL) have higher T_gel_s because these conformations favor more hydrogen bonding between double helices and resist plasticization during heating [11]. Therefore, the structure of amylopectin affects the thermal properties of starch, and starches with more stable amylopectin crystalline regions have higher T_gel_s.

The presence of solutes, such as sugars, also affects the T_gel_. Early theories of why sugars increase the T_gel_ were: sugars decrease the water activity (a_w_) and moisture content [21,22,23]; sugars increase the glass transition temperature (T_g_) of the amorphous fraction [24,25]; and sugar-starch intermolecular interactions stabilize starch [22,26,27,28]. These theories were evaluated using wheat starch and a variety of 19 sweeteners in a recent study by Allan, Rajwa and Mauer [29], who found that T_gel_ increases were correlated to sweetener solution viscosity and the number of exocyclic and equatorial hydroxyl groups on the sweetener, and that T_gel_ increases were not strongly correlated to a_w_ and dry sweetener T_g_s. In a follow-up study by van der Sman and Mauer [30], sweetener solution viscosity was correlated with the volumetric density of intermolecular hydrogen bonds; thus, the T_gel_ increase for wheat starch in sweetener solutions was presumed to be due to sweetener-starch intermolecular hydrogen bond interactions, with a greater number and strength of these interactions resulting in increased T_gel_s.

The effects of sugars on T_gel_ have largely been studied within the scope of a single starch type and without regard to the potential effects of starch granule differences between starch botanical sources and compositions. To expand on our earlier study of the effects of 19 sweeteners on wheat starch T_gel_ [29], the objective of this study was to investigate the effects of different sweeteners on the T_gel_s of six starches with varying granule morphologies (crystal type, percent crystallinity, amylose content, amylopectin chain length distribution, NBbl, and IB-CL) to elucidate the effects of starch botanical sources (and hence structure) on sweetener-starch interactions and resultant T_gel_ elevation.

## 2. Materials and Methods

### 2.1. Materials

Melojel^®^ dent corn starch (dent corn), Amioca waxy corn starch (waxy corn), PenPure^®^ 10 potato starch (potato), Hylon^®^ VII high amylose (≈70% amylose) corn starch (HACS70), and Hylon^®^ V high amylose (≈55% amylose) corn starch (HACS55) were donated by Ingredion Inc. (Westchester, IL, USA), and Aytex^®^ P wheat starch was donated by ADM (Minneapolis, MN, USA) (Table 1). All starches were unmodified and used “as is”. Eleven different sugars and sugar alcohols that are commonly used as food ingredients and/or have minor stereochemical differences of interest for this study were used: glucose, galactose, fructose, and mannose from Acros Organics (Fair Lawn, NJ, USA); trehalose dihydrate from Hayashibara Company (Okayama, Japan); maltose monohydrate from Fisher Bioreagents (Fair Lawn, NJ, USA); isomaltulose monohydrate and isomalt from BENEO-Palatinit Gmbh (Mannheim, Germany); sucrose from Mallinckrodt Chemicals (Phillipsburg, NJ, USA); and maltitol and sorbitol from Alfa Aesar (Ward Hill, MA, USA) (Table 1). Sodium hydroxide (NaOH) was from Sigma-Aldrich (St. Louis, MO, USA), and hydrochloric acid (37%) (HCl) was from Acros Organics. The water used in this study was processed using reverse osmosis, then filtered by a Barnstead E-Pure Lab Water System (Dubuque, IA, USA) to >17.4 milliohm-cm.

### 2.2. Methods

#### 2.2.1. Sweetener Solutions

The 6-carbon (6-C) sweeteners studied consisted of aldoses (glucose, mannose, galactose), a ketose (fructose), and an alditol (sorbitol). Solutions were prepared at 1, 2, 3, and 4 molar (M) concentrations for the 6-C sweeteners except for galactose, for which only 1 and 2 M solutions were used due to solubility limitations. The 12-carbon (12-C) sweeteners consisted of reducing sugars (maltose, isomaltulose), non-reducing sugars (trehalose, sucrose), and sugar alcohols (isomalt, maltitol), and solutions were prepared at 0.5, 1.0, and 1.5 M concentrations, with an additional 2.0 M sucrose solution. The sweetener solutions were prepared in 10 mL volumes in 15 mL centrifuge tubes. Water was added to the sweetener to ~80% of the final volume, then the tube was placed in a heating block at 80 °C for several minutes followed by vortexing on a VWR Vortex Mixer (Lebanon, NJ, USA) and/or slower rotational mixing using a Scientific Industries Roto-Shake Genie (Bohemia, NY) until the sweetener fully dissolved. Upon cooling to near ambient temperature, water was added to reach a final 10 mL volume. Solutions were not used if crystals were visible after overnight storage at ambient conditions.

#### 2.2.2. Gelatinization Temperature (T_gel_)

The gelatinization temperatures (T_gel_s) of the starch-sweetener slurries were measured using a method adapted from Allan, Rajwa and Mauer [29]. Approximately 250 mg of 1:2 *w/w* starch slurries were made by combining 1 part starch with precisely 2 parts solution in 1.5 mL centrifuge tubes, mixing with a stainless steel pin, and vortexing until the slurry appeared to be a homogenous mixture (no visible dry starch). The samples were then capped and stored overnight at ambient temperature (~22 °C). Before analysis, samples were vortexed to re-suspend the starch, and 15 to 20 mg of slurry were transferred into a Perkin Elmer 50 µL pan (BO143017) and hermetically sealed with a lid (BO143003). Due to analysis temperatures exceeding 115 °C and risk of pan failure, the HACS70 and HACS55 samples were loaded into high-pressure Perkin Elmer 50 µL pans (B016-9321) and hermetically sealed with high-pressure lids (B016-9321). Pans were manually transferred into a Perkin Elmer DSC 4000 (Waltham, MA, USA) that was calibrated using water, indium, and zinc. The potato, dent corn, and waxy corn samples were heated from 30 °C up to 100–115 °C at 10 °C/min, while HACS70 and HACS55 samples were heated from 30 °C up to 115–130 °C at 10 °C/min. The T_gel_ of each slurry was measured in triplicate. The T_gel_ was identified as the endothermic event occurring around 60 to 103 °C, and the onset temperature, peak temperature, and enthalpy (∆H) of starch gelatinization were determined using the “peak calculation” function with the “Standard” baseline in Pyris Software (version 10.1.0.0412). T_gel_ peak analysis was performed using data that encompassed ~5 to 10 °C before the peak onset to the post peak heat flow maxima (~2 to 5 °C after the peak end) (Figure S1) while ensuring the peak analysis baseline did not cut through or go under any part of the thermogram. The onset of the T_gel_ peak was calculated by the Pyris software as the intersection of the tangent of the baseline before the peak with the tangent of the inflection of the leading side of the peak (Figure S1). The ∆H of gelatinization was the measured area of the peak, and the reported J/g was of the 1:2 *w/w* starch solution slurry (not adjusted to J/g of dry starch). The onset T_gel_ of HACS70 was calculated using the “onset” function due to lack of a clear end of peak and/or only partial gelatinization occurring within the experimental parameters. This onset was determined by the intersection of two tangents, where the 1st tangent was the slope before gelatinization and the 2nd tangent was the slope between the onset and peak. Examples of DSC analysis for starch in water are shown in Figure S1. Since some samples lacked a clear endotherm peak, the onset T_gel_s were compared further in this study.

The effects of pH on the T_gel_ of potato starch in 1 M glucose solutions were determined using a modified sample preparation method prior to DSC analysis. An initial 150 g sample of a 1:2 *w/w* starch slurry made with 1 M glucose was mixed in a 250 mL beaker, and then the pH was adjusted to 4, 5, 6, 7, 8, 9, and 10 using 1 M glucose solutions that had been pH-adjusted using 1 M NaOH or 1 M HCl. The pH was measured using an Orion pH probe (ThermoScientific, Waltham, MA, USA) calibrated from pH 4 to 10. The pH of the slurry was adjusted rather than the glucose solution to avoid any unaccounted pH buffering from the phosphates in the potato starch. The final starch-to-slurry *w/w* ratio was no longer precisely 1:2 *w/w* due to the addition of the acidified and alkalinized 1 M glucose solutions, but this was not a major concern since the T_gel_ onset in an abundance of solution is not affected by minor starch:solution ratio changes [37]. The slurries were equilibrated overnight, and the T_gel_ of each pH-controlled slurry was then measured in triplicate.

#### 2.2.3. Data Analysis

The effects of sweeteners on the T_gel_ of a starch were compared by grouping the T_gel_s of a starch in sweetener solutions with equal monosaccharide unit concentrations (e.g., 1 M monosaccharide and 0.5 M disaccharide solutions were grouped together), followed by one-way ANOVA and Tukey post-hoc tests (α = 0.05). The effects of a sweetener solution on the T_gel_s of different starches were investigated by comparing ΔT_gel(*i-*0)_s. The ΔT_gel(*i-*0)_ was calculated as the temperature difference between the T_gel_ of a starch in a sweetener solution to that in water. Significant differences of ΔT_gel(*i-*0)_s between starches in a sweetener solution were identified using one-way ANOVA and Tukey post-hoc tests (α = 0.05). The T_gel_s of potato starch in 1 M glucose solutions at pHs 4–10 were also compared using one-way ANOVA and Tukey post-hoc tests (α = 0.05). The associations of categorical sweetener solution properties to effects on T_gel_s were investigated using four-way ANOVA with all possible two-way interactions (α = 0.05). The categorical sweetener solution properties were: monosaccharide unit concentration (e.g., 1 M was 1 M mono- and 0.5 M disaccharide solutions), sweetener size (6-C or 12-C), sweetener type (sugar or sugar alcohol), and if the sweetener was a reducing sugar. The correlation of quantitative starch (Table 1) and sweetener solution properties (Table 2) to the T_gel_s were investigated using linear correlations and *t*-statistics. The tested quantitative starch properties were: percent crystallinity, amylose content (apparent and absolute), average amylopectin chain length, percentage of DP 6–12, percentage of DP 13–24, percentage of DP 25–36, percentage of DP ≥ 37, number of building blocks per cluster (NBbl), and inter-block chain lengths (IB-CL). The quantitative sweetener solution properties were: number of hydroxyl groups for intermolecular H-bonding (N*_OH,s_*), number of equatorial and exocyclic hydroxyl groups, dry glass transition (T_g_), sweetener molar volume, and capacity factor (*K_c_*) (Table 2). These starch and sweetener properties were correlated with the ΔT_gel(*i-*0)_s in sweetener solutions at 3 M monosaccharide unit concentrations (ΔT_gel(3M-0)_) and with the slope of the log_10_ of T_gel_s in respect to molar monosaccharide unit concentrations (log T_gel_s). The significance of the Pearson correlation coefficient (R) was assessed using *t*-statistics:

(1) $$t=\frac{R}{\sqrt{\frac{1-R^{2}}{n-2}}}$$

with *n*−2 degrees of freedom. The significance of crystalline starch type (A or B-type) was evaluated using a two-tailed *t*-test. The effective water contents (*ϕ_w_*,*eff*) of sweetener solutions were extracted from van der Sman and Mauer [30]. All ANOVA tests were performed using IBM SPSS Statistics v. 26.0.00 (Armonk, NY, USA), and correlations and *t*-statistics were calculated using Microsoft Excel 365 (Redmond, WA, USA).

## 3. Results and Discussion

### 3.1. Effects of Sweetener Properties on the Gelatinization Temperatures of Starches

#### 3.1.1. Wheat Starch

**Figure 1:**
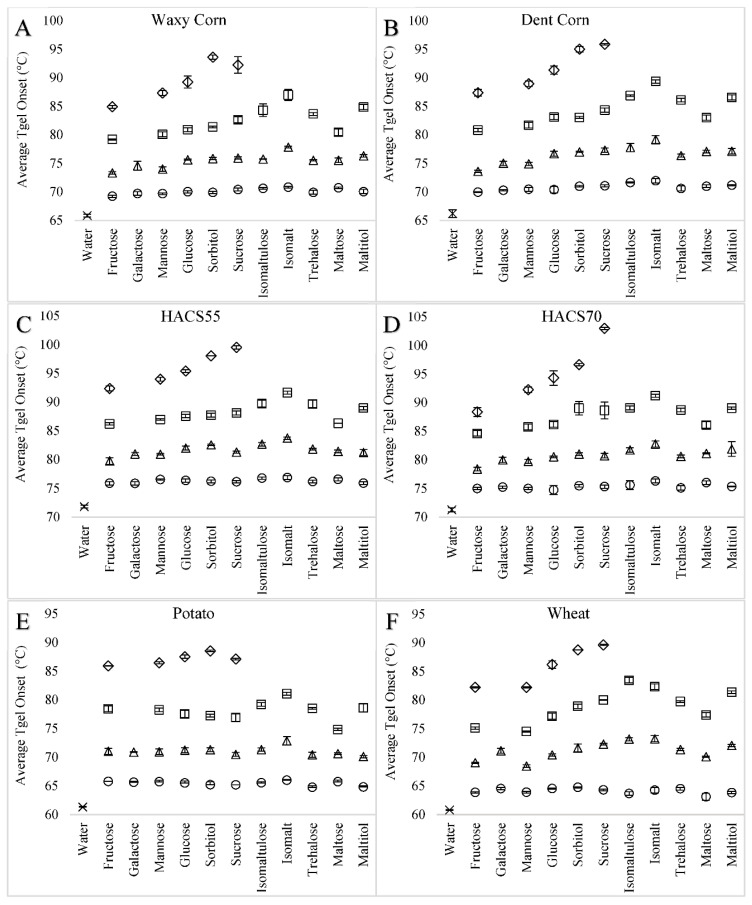
Effects of sweetener type and concentration in solution on the onset T_gel_s of different starches: (**A**) waxy corn, (**B**) dent corn, (**C**) HACS55, (**D**) HACS70, (**E**) potato, and (**F**) wheat starch. Sweetener solutions are grouped by similar solids content: 1 M mono-, 0.5 M disaccharide solutions (○); 2 M mono-, 1 M disaccharide solutions (Δ); 3 M mono- and 1.5 M disaccharide solutions (□); 4 M mono- and 2 M disaccharide solutions (◊); and the control with only water (×). Error bars are 1 standard deviation and *n* = 3.

**Figure 2:**
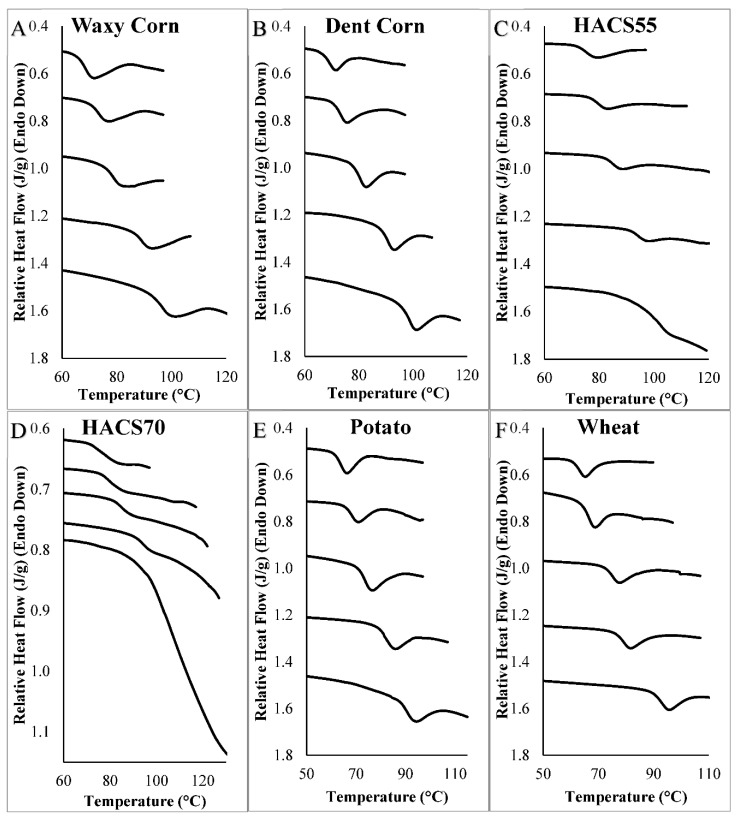
DSC thermograms of 1:2 *w/w* starch slurries in solutions containing different concentrations of sucrose: (**A**) waxy corn, (**B**) dent corn, (**C**) HACS55, (**D**) HACS70, (**E**) potato, and (**F**) wheat starch. Thermograms are shown from top to bottom from: control, 0.5 M sucrose, 1.0 M sucrose, 1.5 M sucrose, and 2.0 M sucrose.

The presence of a sweetener in solution increased the T_gel_ of wheat starch, and increasing sweetener concentrations resulted in further increases in T_gel_ (Figure 1). For example, from a starting point of 60.78 °C in water, the T_gel_ of wheat starch increased by 3.54, 11.50, 19.20, and 28.86 °C in 0.5, 1.0, 1.5, and 2.0 M sucrose solutions (Figure 1 and Figure 2, Table S1). The type of sweetener also had a significant effect on the wheat starch T_gel_. In 3 M monosaccharide unit solutions, fructose and mannose increased the T_gel_ the least, whereas isomaltulose followed by sucrose, isomalt, maltitol, and trehalose increased the T_gel_ the most (Figure 1 and Table S1). The 12-C sweeteners typically increased the wheat starch T_gel_ more than 6-C sweeteners when compared at equal monosaccharide unit concentrations, except for sorbitol which increased the T_gel_ more than maltose (Table 3 and Table S1). The gelatinization endotherm of wheat starch remained as a single unimodal peak in the presence of sweeteners (Figure 2). Therefore, unlike what is found in low moisture conditions where additional thermal energy is needed to fully melt amylopectin, there was no peak separation between G (solution mediated melting of crystallites that is independent of moisture content) and M1 (melting of remaining crystallites that is dependent on moisture content) [37,44]—increasing sweetener concentrations simply increased the T_gel_ endotherm to a higher temperature.

Sugar alcohols tended to increase the wheat starch T_gel_ more than the sugar counterparts at the same molar concentrations (e.g., Figure 1 and Table S1, sorbitol vs. glucose), except between isomalt and isomaltulose. In Allan, Rajwa and Mauer [29], the wheat starch T_gel_s in xylitol solutions were also significantly higher than in xylose solutions and sugar alcohols were speculated to increase the wheat starch T_gel_ more than sugars by potentially forming more H-bonds within the amorphous regions of native starch. Sugar alcohols may form more sweetener-starch interactions because they have an open structure [45], greater molar volume, and greater number of exocyclic hydroxyl groups (Table 2) than sugars.

The quantitative sweetener properties in Table 2 were also compared to the sweetener effects on wheat starch T_gel_ in 3 M monosaccharide unit sweetener solutions (ΔT_gel(3M-0)_) and the slope of the log of T_gel_s with respect to the sweetener molar monosaccharide unit concentration (log T_gel_). Significant correlations (*p* < 0.10) were found between T_gel_ increases in sweetener solutions and both the number of equatorial and exocyclic hydroxyl groups on the sweetener and the sweetener molar volume, whereas the correlations of T_gel_ with N*_OH,s_*, T_g_, and *K*_c_ were not significant (Table 4). The correlation between wheat starch T_gel_ and the number of equatorial and exocyclic hydroxyl groups suggests that sweetener stereochemistry influences the T_gel_ increase. Similar findings were found for sweetener-water interactions, for which there was a strong correlation between the number of equatorial hydroxyl groups and dynamic hydration number of a sweetener [46]. The sweetener stereochemistry affects intermolecular H-bonding tendencies because exocyclic and equatorial hydroxyl groups are more reactive than the axial hydroxyl groups [47], and the strength and number of sweetener-starch interactions affect the T_gel_ increase [29]. The sweetener molar volume was also significantly correlated to the effects on wheat starch T_gel_ (Table 4), as were the sweetener solution viscosities (R ≥ 0.96, Table S2). Overall, 12-C sweeteners had larger solute radii and increased the T_gel_ more than 6-C sweeteners, and sugar alcohols had larger radii and increased the T_gel_ more than their sugar counterparts (Table 2 and Table 4). Conceptually, sweeteners with larger solute radii can form longer H-bond bridges between chains in the amorphous regions of native starch and therefore have a greater stabilizing effect.

Several sweetener properties did not positively correlate with wheat starch T_gel_, including N*_OH,s_*, T_g_, and capacity factor (*K_c_*) (TableS 2 and 4). In Allan, Rajwa and Mauer [29], the direct effect of sweetener T_g_ on the T_gel_ was doubted due to the presence of extreme outliers and evidence the T_g_ of native starch is well below the T_gel_ [7,48]. Since N*_OH,s_* was derived from the T_g_ of the sweetener [49], it was therefore not surprising there was no correlation of T_gel_ with either T_g_ or N*_OH,s_*. However, the effective water content (*φ_W_, eff*), which is directly related to volumetric density of intermolecular hydrogen bonds of the sweetener solution and calculated using N*_OH,s_* [30], was still highly correlated to the starch T_gel_ (Figure S2). A low *K_c_* was associated with greater hydrophobicity in a C-18 HPLC column, but the applicability of *K_c_* for determining sweetener effects on starch T_gel_ was limited as it was not correlated to sweetener hydration numbers, NMR relaxation, terahertz spectroscopy, or viscosity [40]. Poor correlations between sweetener solution a_w_ and T_gel_ increase have been found [29,30], and there is no chemical need for water during starch gelatinization since starch can gelatinize in pure glycerol [28].

#### 3.1.2. Waxy, Dent, and High Amylose Corn Starches

**Figure 3:**
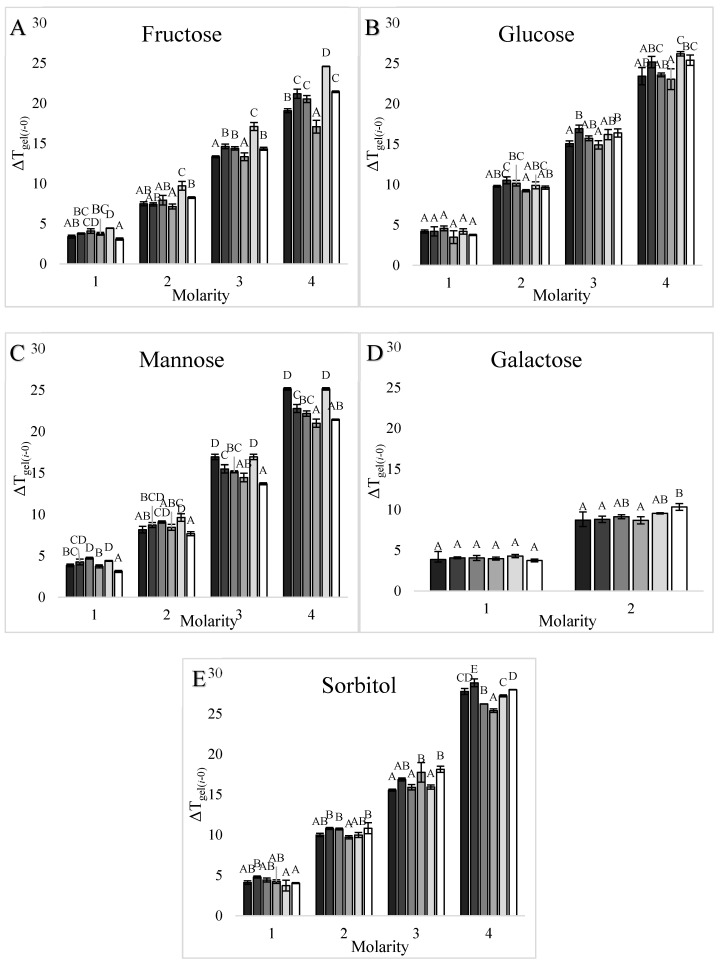
Effects of increasing concentrations (1-4 M) of 6-carbon sweeteners in solution on the onset T_gel_ of different types of starch: (**A**) fructose, (**B**) glucose, (**C**) mannose, (**D**) galactose, and (**E**) sorbitol. Starch from left to right as waxy corn (█), dent corn (█), HACS55 (█), HACS70 (█), potato (█), and wheat starch (█). The increase in the onset T_gel_ in the sweetener solution compared to the onset T_gel_ in water (ΔT_gel(*i-*0)_) is plotted. Error bars are 1 standard deviation, *n* = 3, and the capital letters indicate significant differences between T_gel_s.

**Figure 4:**
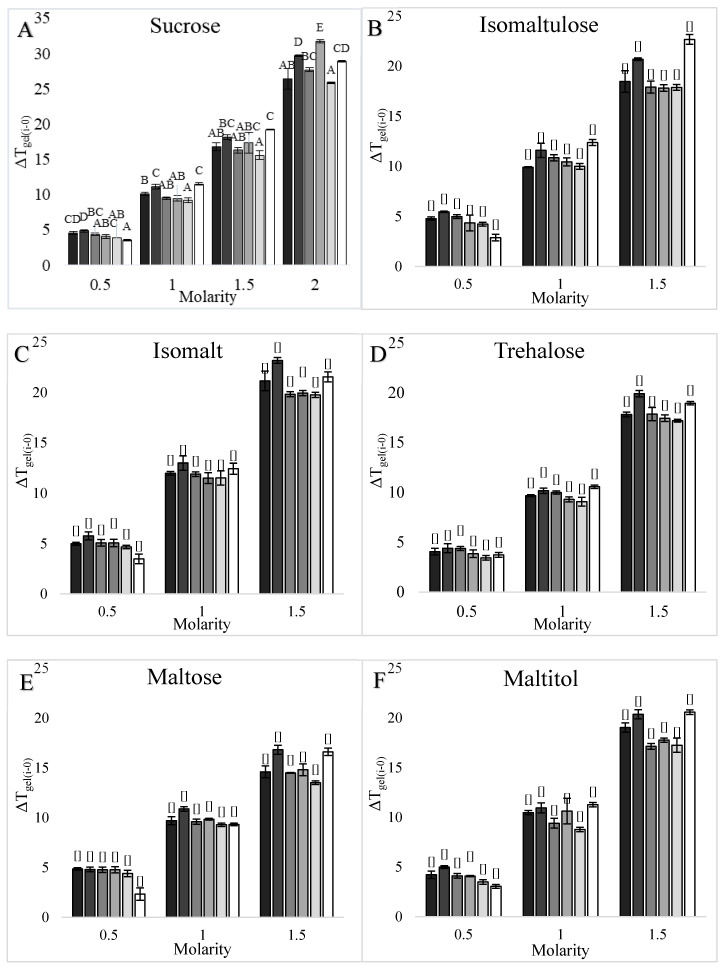
Effects of increasing concentrations (0.5–2.0 M) of 12-carbon sweeteners in solution on the onset T_gel_ of different types of starch: (**A**) sucrose, (**B**) isomaltulose, (**C**) isomalt, (**D**) trehalose, (**E**) maltose, and (**F**) maltitol. Starch from left to right as waxy corn (█), dent corn (█), HACS55 (█), HACS70 (█), potato (█), and wheat starch (█). The increase in the onset T_gel_ in the sweetener solution compared to the onset T_gel_ in water (ΔT_gel(*i-*0)_) is plotted. Error bars are 1 standard deviation, *n = 3*, and the capital letters indicate significant differences between T_gel_s.

As was found for wheat starch T_gel_, increasing sweetener concentrations also significantly increased the T_gel_ of corn starches (Figure 1), with differences found between sweetener types. For example, at 3 M monosaccharide unit concentration, isomalt increased the T_gel_ of corn starches the most and fructose increased the T_gel_ the least (Tables S3–S6). Significant differences were also found between corn starch types (Figure 3 and Figure 4), with dent corn starch generally exhibiting the greatest T_gel_ increase (ΔT_gel(*i-*0)_) in the presence of sweeteners compared to the other corn starches. Exceptions were the ΔT_gel(*i-*0)_s of waxy corn in 3 and 4 M mannose solutions and HACS70 in 2 M sucrose solutions, which were greater than the ΔT_gel(*i-*0)_s of dent corn (Figure 3 and Figure 4). The HACS70 T_gel_ in 2 M sucrose solution was exceptionally high, likely due to the unique shape of the thermogram (Figure 2). The substantial shift in the baseline for this sample may be the onset of amylopectin hydrate dehydration or a T_g_ event.

Similar to what was found for wheat starch T_gel_s, the categorical sweetener properties that were significantly correlated to the T_gel_s of all corn starches were sweetener concentration and size. A difference between corn and wheat starch behaviors was that the sweetener type (sugar or sugar alcohol) was significant for the T_gel_s of all corn starches but not for wheat starch T_gel_s (Table 3). Corn starch T_gel_s in 12-C sweetener solutions were generally greater than in 6-C sweetener solutions within the same monosaccharide unit concentration grouping; however, the T_gel_s in some 6-C sweetener solutions, such as sorbitol, were significantly greater than in some 12-C sweetener solutions, such as maltose (Tables S3–S6). The sweetener type (sugar or sugar alcohol) was significant for all corn starches (Table 3), with the corn starch T_gel_s in sugar alcohol solutions (sorbitol, isomalt, and maltitol) equal to or greater than in the counterpart sugar solutions (glucose, isomaltulose, and maltose, respectively) (Tables S3–S6). Baek, Yoo and Lim [50] also observed that corn starch T_gel_s in sugar alcohol solutions were greater than in sugar solutions.

The quantitative sweetener properties that were significantly correlated to the T_gel_ elevation of corn starches were the numbers of equatorial and exocyclic hydroxyl groups and molar volumes (*p* < 0.10), except not the molar volume with log T_gel_s of HACS55 (Table 4). Similar to what was found for wheat starch T_gel_, the N*_OH,s_*, T_g_, and *K_c_* of sweeteners were not significantly correlated to the sweetener effect on corn starch T_gel_, while the *φ_W_, eff* and solution viscosity were highly correlated to the corn starch T_gel_s (Figures S3–S6 and Table S2). Sweeteners that formed more H-bonds with neighboring molecules and had larger solute radii increased the T_gel_s of corn starches to a greater extent.

#### 3.1.3. Potato Starch

Similar to wheat and corn starches, increasing sweetener concentrations increased the T_gel_ of potato starch; however, potato starch T_gel_ responded differently to some sweeteners compared to the other starches (Figure 1 and Table S7). In 3 M monosaccharide unit concentration solutions, the highest potato starch T_gel_ was in a 1.5 M isomalt solution and the lowest was in a 1.5 M maltose solution (Table S7), which was different from the other starches for which the lowest T_gel_s were in 3 M fructose solutions (Tables S1–S5). Fewer differences were found in potato starch T_gel_s, compared to wheat and corn starches, with respect to the different effects of 6-C and 12-C sweeteners on T_gel_. The 6-C sweeteners tended to elevate the ΔT_gel(*i-*0)_ of potato starch more than ΔT_gel(*i-*0)_s of other starches; however, the 12-C sweeteners did not elevate the ΔT_gel(*i-*0)_ of potato starch as much as the ΔT_gel(*i-*0)_s of the wheat and corn starches. Thus, the T_gel_ of potato starch was one of the most affected by 6-C sweeteners (Figure 3) and least affected by 12-C sweeteners (Figure 4) compared to the T_gel_s of wheat and corn starches.

Unlike for other starch types, none of the numerical sweetener factors were significantly correlated to the potato starch T_gel_ (Table 4); however, the number of equatorial and exocyclic hydroxyl groups and molar volumes of 6-C sweeteners were significantly correlated with log T_gel_s. When correlating log T_gel_s of potato starch with only 6-C sweetener properties (excluding 12-C sweetener properties), the correlation with the number of equatorial and exocyclic hydroxyl groups increased from R = 0.024 to R = 0.996 (*p* < 0.05), and the correlation with the molar volume increased from R = 0.340 to R = 0.945 (*p* < 0.10). The log T_gel_ of potato starch in galactose solutions was excluded from these correlations due to low galactose solubility resulting in a limited number of data points (only 1 and 2 M). The significant correlations of potato starch log T_gel_ with 6-C sweetener molar volumes and the number of equatorial and exocyclic OHs suggest the mechanism by which 6-C sweeteners increased the T_gel_ of potato, wheat, and corn starches was similar. None of the 12-C sweetener properties were correlated with the log T_gel_s of potato starch, attributed to a potential size limiting effect that altered the sweetener-starch interactions of 12-C sweeteners in the potato starch granule (discussed in detail later).

### 3.2. Effects of Starch Properties

When comparing the effects of sweeteners on the T_gels_ of starches from different botanical sources (Figure 3 and Figure 4), significant differences in the ΔT_gel(*i-*0)_ between starches were found. Differences were also found in the significant sweetener factors associated with the T_gel_s (Table 3 and Table 4) of these starches. Therefore, sweeteners affected the T_gel_s of starches from different botanical sources differently, and thus it was of interest to explore how these differences were influenced by starch composition, architecture, and morphology.

#### 3.2.1. Amylose Content

The starches evaluated had varying amylose compositions ranging from ≈0% amylose in waxy corn starch up to 68% apparent amylose in HACS70 (Table 1). Within the corn starch sources, the effects of sweeteners on the ΔT_gel(*i-*0)_s were not consistent with the amylose contents. For example, the dent corn ΔT_gel(*i-*0)_s were greater than the waxy corn ΔT_gel(*i-*0)_s, except in mannose solutions; however, the ΔT_gel(*i-*0)_s of HACS55 and HACS70, which contained even more amylose, were also less than dent corn (Figure 3 and Figure 4). When evaluating all starch sources, there were no significant correlations (*p* < 0.10) between the ΔT_gel(3M-0)_ or log T_gel_s of a sweetener and the amylose (apparent and absolute) contents of any of the starches (Table S8).

When comparing only waxy and dent corn starches, the presence of amylose increased the ΔT_gel(*i-*0)_ in the presence of sweeteners. The interspersed amylose in the amorphous regions of native starch may have allowed for a greater stabilization effect from sweetener H-bond bridges. However, this trend did not persist with further increases in amylose content, perhaps because additional starch structural changes were present. The high amylose corn starches have much longer amylopectin branch lengths, greater IB-CLs, and are in the B-type crystalline form; while waxy and dent corn have more comparable amylopectin structures (Table 1).

Other studies have found varying relationships between T_gel_ and amylose content: some studies suggest starches with greater amylose contents have higher T_gel_s [51,52], others report no effect on T_gel_ [53,54,55,56], and yet others report lower T_gel_s for starches with higher amylose contents [17]. It has also been proposed that the type of amylose affects the T_gel_ behavior: lipid complexed amylose increases the T_gel_ [19]. Salts have been reported to affect the T_gel_ and ΔH of gelatinization of waxy and normal corn starches in a similar manner [57], thus amylose content made little difference. Therefore, the lack of correlations between the T_gel_ increase in sweetener solutions and the amylose contents of starches (Table S8) suggest amylose contents did not greatly affect the sweetener-starch interactions that increase the T_gel_.

#### 3.2.2. Amylopectin Architecture

The starches in this study had varying amylopectin architectures (Table 1). HACS55 and HACS70 had longer amylopectin chain lengths, smaller percentages of short DP 6–12 amylopectin chains, higher percentages of DP 25-–36 and DP ≥37 amylopectin chains, lower NBbls, and higher IB-CLs (Table 1), and the T_gel_s of these starches were least affected by sweeteners (Figure 3 and Figure 4). The T_gel_s of wheat and dent corn starches were more affected by sweeteners (Figure 3 and Figure 4), and these starches had shorter amylopectin chain lengths, higher ratios of DP 6–12 chains, lower percentages of DP 25-–36 and DP ≥37 amylopectin chains, higher numbers of NBbls, and lower IB-Cls. When comparing all starches in this study, the percentages of DP 25-–36 and DP ≥37 chains and the average amylopectin chain lengths of starches were negatively correlated with T_gel_ elevations in different sweeter solutions (Table 5). In contrast, the T_gel_ elevations of starches in sweetener solutions were positively correlated with higher percentages of DP 6–12 amylopectin chains (Table 5). Therefore, the T_gel_s of starches with higher percentages of short amylopectin chains were more affected by sweeteners than the T_gel_s of starches with longer amylopectin chains. The T_gel_ increases for starches in several sweetener solutions were also positively correlated with the NBbl of starches and negatively correlated with IB-CLs of starches (Table 6), suggesting that amylopectin fine structure also influenced the sweetener-starch interactions.

The architecture of amylopectin has been associated with the thermal properties of starch, attributed to the stability of the starch crystallites [14,15,19,20,55,58,59,60]. Starches with higher ratios of short amylopectin chains tend to have lower T_gel_s because these short chains are too short to crystallize and also act as crystal defects [15,19,20]. Therefore, the starches with higher percentages of DP 6–12 amylopectin chains (e.g., dent corn and wheat starches were 17.9 and 19.0% DP 6–12, respectively) have less stable starch crystallites, and sweeteners presumably formed sweetener-starch interactions that enhanced the crystallite stability and resulted in a greater T_gel_ elevation. Starches with smaller ratios of DP 6–12 amylopectin branches (e.g., HACS55 and HACS70 were 9.7 and 8.5% DP 6–12, respectively) had T_gel_s that were less affected by sweeteners because there were fewer crystal defects. The negative linear correlations between T_gel_ increases in sweetener solutions and average amylopectin chain lengths, percentages of DP 25-–36, and percentages of DP ≥ 37 (Table 5) suggest the T_gel_s of starches with longer amylopectin branches are less affected by sweetener solutions. The DP 6–12, 13–24, 25–36, and ≥37 amylopectin chains fractions have been defined as A (fa), B1 (fb_1_), B2 (fb_2_), and B3 (fb_3_) chains and span through 1, 1, 2, and 3 crystalline amylopectin clusters, respectively [61,62]. The B2 (DP 25-–36) and B3 (DP ≥37) amylopectin branches span through multiple clusters and are long enough to make relatively defect-free crystallites. Crystallites that have fewer crystal defects and are covalently linked to one another are already fairly stable, thus sweeteners in solution have a lower potential for elevating the T_gel_ because there are fewer possibilities for stabilizing sweetener-starch intermolecular interactions. There were also no significant correlations between T_gel_ increases in sweetener solutions and percentages of B1 (DP 13-–24) chains, which could be due to the intermediate length between stabilizing and destabilizing amylopectin chain lengths.

The fine structure of amylopectin also affects the T_gel_, with lower NBbl and higher IB-CL values associated with higher T_gel_s [11]. There were significant correlations with NBbl and IB-CL of starches and the effects of sweeteners increasing the T_gel_s (Table 6). With fewer building blocks in a cluster (NBbl) and greater spacing between building blocks (IB-CL) allowing for more molecular flexibility, the double helices in the crystalline amylopectin clusters are able to form more hydrogen bonds and a stronger crystal structure [11]. Therefore, native starches with more blocks per cluster and shorter spacing between building blocks have less stable crystallites, and thus sweetener-starch interactions have a greater stabilizing effect (Table 5). For example, the T_gel_s of wheat and dent corn starches increased the most in sweetener solutions (Figure 3 and Figure 4) and these starches had the greatest number of building blocks per cluster (NBbl) and shorter IB-CLs (Table 1). In contrast, the T_gel_s of high amylose corn starches had the smallest T_gel_ increases in sweetener solutions (Figure 3 and Figure 4) and had the highest IB-CLs and lower NBbl values. Again, the T_gel_s of starches with more stable crystals were less affected by sweeteners.

Also of note, sweeteners that increased the T_gel_ the most (isomalt, maltitol, and isomaltulose (Figure 1)) tended to have the strongest correlations between T_gel_ increase and amylopectin lengths, NBb1, and IB-CL (Table 5 and Table 6). It was postulated this occurred because these sweeteners had the greatest stabilizing effect due to the larger molar volumes and higher numbers of equatorial and exocyclic hydroxyl groups (Table 2), and starches with the greatest potential for sweetener-starch stabilizing interactions had T_gel_s that were more affected by these sweeteners.

In summary, amylopectin structure affected the extent to which sweeteners increased the T_gel_. The T_gel_s of starches that had less stable crystallites, due to shorter amylopectin chain lengths and sterically hindered double helical clusters, were more affected in sweetener solutions than starches with more stable crystalline regions. Since sweetener-starch interactions drive the T_gel_ increase, starches that have more regions to be stabilized (e.g., wheat and dent corn starches as shown in Table 5 and Table 6) had greater T_gel_ increases in the presence of sweetener solutions.

#### 3.2.3. Amylopectin Crystalline Structure

Starches with A-type and B-type amylopectin crystalline structures were used in this study: waxy corn, dent corn, and wheat starch were the A-type polymorphic form while HACS55, HACS70, and potato starches were the B-type polymorphic form (Table 1). The T_gel_s of A-type starches increased more in sweetener solutions than the T_gel_s of B-type starches (Table S9) based on comparing the averages of log T_gel_s and the ΔT_gel(3M-0)_s in A and B-type starches. However, the ΔT_gel(*i-*0)_s of dent corn (A-type), wheat (A-type), and potato in 6-C solutions (B-type) starches were the most affected by sweeteners, while the ΔT_gel(*i-*0)_s of waxy (A-type), potato (B-type) in 12-C solutions, and high amylose corn starches (B-type) were the least affected (Figure 3 and Figure 4). Thus, there did not appear to be a consistent trend in ΔT_gel(*i-*0)_ response to sweeteners in solution based solely on amylopectin crystal polymorph. A confounding factor was likely that starches with longer amylopectin chains form B-type crystal structures (Table 1) and the T_gel_s of starches with longer amylopectin branches were less affected by sweeteners (e.g., high amylose corn starches in Figure 3 and Figure 4). In theory, the crystalline form should not affect the influence of sweeteners on T_gel_, because sweeteners increase the T_gel_ through intermolecular interactions in the amorphous regions of starch [29].

#### 3.2.4. Percent Crystallinity

No correlation was found between the percent crystallinity of starches and the effects of sweeteners on the T_gel_ increase (Table S8). For example, the high amylose corn and wheat starches were all ~20% crystalline, yet the T_gel_ of wheat starch was more influenced by sweeteners than the T_gels_ of the high amylose corn starches (Figure 3 and Figure 4). Even though the sweetener-starch interactions that stabilize the granule and increase the T_gel_ occur in the amorphous regions [29], perhaps the amount or distribution of the amorphous regions were not limiting factors within this experimental space, since the native starches in this study were all >50% amorphous.

#### 3.2.5. Potato Starch—The Anomaly

There were similar sweetener effects on ΔT_gel(*i-*0)_ trends between starches from different botanical sources (Figure 3 and Figure 4) with few exceptions other than for potato starch. Potato starch was also the only starch that had a T_gel_ not affected by the sweetener size (Table 2), since potato starch T_gel_s in 6-C sweetener solutions were not significantly different from T_gel_s in 12-C sweetener solutions. It has been reported that 12-C sweeteners (disaccharides) increase the T_gel_ of starch more than 6-C sweeteners (monosaccharides) for other starch botanical sources [22,29,50]. Therefore, the unique aspects of potato starch were explored to elucidate why the sweetener effects on the T_gel_ elevation behavior of potato starch were different than for other starches.

(1) Size Exclusion within the Granule

A speculation as to why 12-C sweeteners were not as effective at raising the T_gel_ of potato starch compared to elevating the T_gel_s of starches from other botanical sources was due to some sort of size exclusion, wherein the diffusion of 12-C sweeteners throughout the potato starch granule was affected differently than diffusion in other starch types. The molecular size exclusion could be due to: (1) the spacing of amylopectin branch points, and/or (2) the spacing between blocklets in the crystalline growth rings. In potato starch, the amylopectin branch length from the backbone to the crystalline double helix is ~3 glucosyl units long, much shorter than for waxy rice, tapioca, and wheat starches that have branch lengths of 6, 7, and 7 glucosyl units long, respectively [63]. This short distance between the backbone to the crystalline helix in potato starch creates a smaller region for sweetener-starch interactions in the amorphous lamella, which potentially creates steric hindrance or spatial challenges for a DP 2 sweetener in a space that is ~DP 3 in size. This amylopectin branch length between the backbone and double helix is not to be confused with amylopectin lamella spacing, which is consistently ~9 nm between starch sources [5]. Another possible size exclusion point is the space between the crystalline blocklets in the crystalline growth rings. The blocklets in potato starch are much larger (200–500 nm) than the blocklets in wheat starch (80–120 nm), which reduces the porosity of potato starch [6,64]. This lower porosity in the crystalline growth ring may restrict the diffusion of 12-C sweeteners and thus affect the localized concentration of sweetener solutions in the amorphous regions of potato starch.

(2) Phosphorous Content

**Figure 5:**
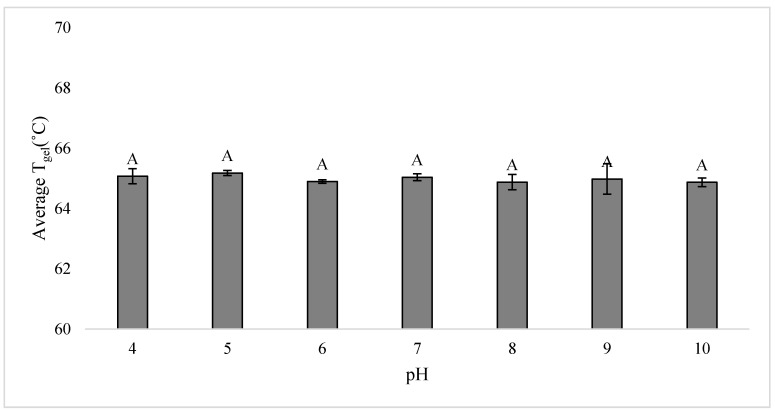
The onset T_gel_s of potato starch in 1 M glucose solutions at pHs 4, 5, 6, 7, 8, 9, and 10. Error bars are 1 standard deviation, *n* = 3, and the capital letters indicate significant differences in T_gel_s.

Another unique aspect of potato starch is that it contains phosphate monoesters. The phosphate monoesters cause potato starch to be affected by electrolytes [65] and have a lower T_gel_ compared to other starches with similar amylopectin structures, because the crystallites are destabilized when the phosphate groups repel from each other during heating [64,66]. The phosphate groups are predominantly (74–78%) in the amorphous regions [67]; however, phosphate content has been both positively correlated [67] and not correlated [20,56] to the potato starch T_gel_. It is important to note that the positive correlation of phosphate content and T_gel_ could have been confounded with the effects of longer amylopectin chains [67], which also elevate T_gel_. Since phosphates are ionizable groups, it was assumed the p*K*_a_s of potato starch were similar to the p*K*_a_s of phosphoric acid: there would be no p*K*_a3_ at 12.67 due to the ester linkage with starch, p*K*_a1_ at 2.21, and p*K*_a2_ at 7.21 (p*K*_a_ values from Weast [68]). A similar assumption was made by Marsh and Waight [69], but the p*K*_a_s of potato starch have also been assumed to be similar to those of glucose 6-phosphate at p*K*_a1_ ≈ 0.94 and p*K*_a2_ ≈ 6.11 [70,71]. Regardless, the T_gel_s of potato starch in 1 M glucose solutions with pHs ranging from 4 to 10 were the same, at ~65 °C (Figure 5). The lack of T_gel_ changes at pHs above and below p*K*_a2_ in the presence of a sweetener suggests that the phosphate monoesters do not influence the sweetener-starch interactions to an extent that would affect the T_gel_. The phosphate monoesters may not affect sweetener-starch interactions because there is a relatively low concentration of phosphate groups: one per ~317 glucosyl units [72]. Another explanation could be that the phosphate monoesters are primarily located on long B chains that are greater than DP 20 [72], and DP > 18 amylopectin branches are the stabilizing portions in potato starch since they yield more stable crystals and higher T_gel_s [20]. The T_gel_ of yam starch, which has about one-seventh of the amount of phosphate monoesters compared to potato starch [73], was also unaffected by changes in pH [74].

Therefore, the most likely reason potato starch T_gel_s exhibited different trends in the presence of 12-C sweeteners compared to T_gel_s of starches from other botanical sources is that the sweetener diffusion throughout the amorphous regions of the potato starch granule was restricted more than in other starch types, thereby limiting the amount of 12-C sweetener-starch interactions in native potato starch.

## 4. Conclusions

The effects of 6-C and 12-C sweeteners on the T_gel_s of six different starches were investigated. The T_gel_s of all starches increased in sweetener solutions compared to T_gel_s in water, with greater T_gel_ increases as sweetener concentration increased; however, the magnitude of the increase in T_gel_ varied between different types of sweeteners and starches. The starch T_gel_ was increased more by sweeteners that had larger molar volumes and higher numbers of equatorial and exocyclic hydroxyl groups (e.g., sugar alcohols and 12-C sweeteners). Sweeteners with these properties formed more sweetener-starch interactions, stabilizing the amorphous starch regions, and thereby increased the T_gel_ to a greater extent. Sweetener traits that were not associated with increases in starch T_gel_ were: dry T_g_, *K_c_*, N*_OH,s_*, and whether or not the sweetener was a reducing sugar. The amylopectin structure of starches also affected the T_gel_ increase in sweetener solutions. The T_gel_s of starches with higher percentages of A-chains (DP 6–12) increased the most in sweetener solutions, whereas the T_gel_s of starches with higher percentages of B2 (DP 25-–36) and B3 (DP ≥37) amylopectin chains increased less in sweetener solutions. The amylopectin fine structure also influenced the effects of sweetener solutions on the T_gel_, where starches with fewer building blocks per cluster and longer inter-block chain lengths formed more stable crystals and the T_gel_s were less affected. A-type starches were more affected by sweeteners than B-type starches, but this may be confounded with B-type starches having longer amylopectin chains. Starch traits that were not associated with T_gel_ increases in the presence of sweeteners included: amylose content and percent crystallinity. The effects of sweeteners on the T_gel_ elevation of potato starch were unique, because the potato starch T_gel_ was one of the most affected by 6-C sweeteners and the least affected by 12-C sweeteners. This was attributed to a size exclusion phenomenon that altered 12-C sweetener diffusion into the potato starch granules and was not due to ionic effects of the phosphate monoesters. In summary, starches with less stable crystalline regions are more susceptible to sweetener-starch stabilizing effects and exhibited greater increases in T_gel_ in the presence of sweeteners. Native starches with structures that limit sweetener-starch interactions, such as the more stable crystalline regions in high-amylose corn starches or the unique structures in potato starch that limit diffusion of 12-C sweeteners, exhibit smaller increases in T_gel_. However, sweeteners with molecular conformations that were favorable for intermolecular interactions increased the T_gel_ the most, regardless of starch architecture. This study has shown the different T_gel_ elevation effects of sweeteners on multiple starch botanical sources and provided insights into structural reasons for these differences, information that is useful for improving the understanding of structure-function relationships and behaviors of starches in formulations containing different sweeteners.

## Figures and Tables

**Table 1:** Physical and chemical properties of the starches used in this study.

				Amylose Content [2]		Average	% Distribution [2]						Granule	Phosp-
Starch	T_gel_ Onset (°C) ^†^	Percent Crystallinity ^‡^	Crystal Type [2]	Apparent	Absolute	Chain Length (DP) [2]	DP 6–12	DP 13–24	DP 25–36	DP ≥ 37	NBbl	IB-CL	Size (μm) [1]	Horus (%DS) [1]
Waxy corn	65.84 ± 0.25 B	41.8 [31]	A	<1%	<1%	23.5	17.0	49.4	17.1	16.5	5.2 [32]	6.2 [32]	2–30	0.00
Dent corn	66.19 ± 0.65 B	30.3 [31]	A	29.4	22.5	24.4	17.9	47.9	14.9	19.3	6.2 [33]	6.8 [33]	2–30	0.00
HACS55	71.81 ± 0.25 C	19.5 [31]	B	52	27.3	28.9	9.7	43.9	20.3	26.1	5.4 [33]	9.1 [33]	2–24	0.00
HACS70	71.27 ± 0.37 C	20.7 [34]	B	68	40.2	30.7	8.5	40.7	21.3	29.5	5.6 [33]	8.9 [33]	2–24	0.00
Potato	61.32 ± 0.19 A	45.5 [34]	B	36	16.9	29.4	12.3	43.3	15.5	28.9	3–5 [35]	7–8 [35]	5–100	0.08
Wheat	60.78 ± 0.09 A	22.8 [34]	A	28.8	25.8	22.7	19.0	41.7	16.2	13.0	6.2–6.3 [36]	6.4–6.5 [36]	2–55	0.00

^†^ T_gel_s in water. Capital letters indicate statistical groupings. ^‡^ Percent crystallinity data were from reported % crystallinity values calculated using X-ray diffractograms. DP: degree of polymerization.

**Table 2:** Properties of the sweeteners used in this study.

Sweetener	Number of Carbons	Sweetener Type	Reducing Sugar	Glycosidic Linkage [38]	Number of OH Groups for Inter-Molecular H-Bonding [30]	Calculated Equatorial and Exo-Cyclic OHs in Solution [29]	Dry T_g_ (°C) [39]	Molar Volume (cm^3^/mol)	Capacity Factor (*K_c_*) [40]
Fructose	6	Sugar	Yes	NA	3.98	2.8	15.16 ± 0.11	110.4 ± 0.4 [39]	0.029
Mannose	6	Sugar	Yes	NA	4.05	3.3	35.91 ± 0.10	111.7 ± 0.5 [39]	0.026
Galactose	6	Sugar	Yes	NA	3.95	3.6	31.92 ± 0.47	111.9 ± 0.3 [39]	−0.006
Glucose	6	Sugar	Yes	NA	3.98	4.6	38.30 ± 0.01	112.2 ± 0.4 [39]	0.016
Sorbitol	6	Sugar Alcohol	No	NA	3.21	6	−1.54 ± 0.71	119.9 [41]	0.012
Sucrose	12	Sugar	No	αGlc*p*(1 → 2)βFru*f*	4.48	6	59.36 ± 0.56	210.2 ± 0.8 [39]	0.47
Isomaltulose	12	Sugar	Yes	αGlc*p*(1 → 6)Fru	4.75	5.2	60.56 ± 0.61	219.5 [42]	0.177
Isomalt	12	Sugar Alcohol	No	αGlc*p*(1 → 6)Sor & αGlcp(1 → 6)Mtl	4.69	9	58.73 ± 1.63	NA	0.1260.143
Trehalose	12	Sugar	No	αGlc*p*(1 → 1)αGlc*p*	7.72	8	117.51 ± 2.01	206.9 ± 0.5 [39]	0.128
Maltose	12	Sugar	Yes	αGlc*p*(1 → 4)Glc	5.74	7.4	48.99 ± 3.83	208.8 ± 0.8 [39]	0.195
Maltitol	12	Sugar Alcohol	No	αGlc*p*(1 → 4)Sor	4.33	9	46.40 ± 0.11	215.36^7^ [43]	NA

NA: not available.

**Table 3:** Analyses of sweetener solution properties that influenced the T_gel_ onsets of waxy corn, dent corn, HACS55, HACS70, potato, and wheat starch comparisons of *p*-values from four-way ANOVA are shown. Sweetener concentrations groupings were by monomeric molar concentrations (e.g., 1 M is 1 M mono- and 0.5 M disaccharide solutions), size groupings were 6- (e.g., glucose, sorbitol) and 12-carbon sweeteners (e.g., sucrose, isomalt); and reducing sugar groupings were reducing (e.g., glucose, isomaltulose) and nonreducing sweeteners (e.g., sucrose, sorbitol). Significant factors and interactions are shown in bold font (α = 0.05).

	*p*-Values						
Starch	Sweetener Concen-Tration	Size (6 OR 12-C)	Type (Sug./Sug. Alc.)	Reducing Sugar (Red./Nonred.)	Conc.* Size	Conc.* Type	Conc.* Red.
Waxy corn	**<0.001**	**<0.001**	**<0.001**	0.981	**0.001**	0.060	0.660
Dent corn	**<0.001**	**<0.001**	**<0.001**	0.428	**<0.001**	0.198	0.948
HACS55	**<0.001**	**<0.001**	**0.004**	0.913	0.070	0.401	0.358
HACS70	**<0.001**	**<0.001**	**<0.001**	0.899	0.196	0.403	0.042
Potato	**<0.001**	0.661	**0.003**	0.133	0.911	0.423	0.671
Wheat	**<0.001**	**0.001**	0.118	0.513	**<0.001**	0.302	**0.005**

*: A standard for referring to interaction effects between the terms.

**Table 4:** Comparisons of correlation coefficients (R) of slopes of log T_gel_ vs. sweetener concentration (abbreviated log T_gel_ in table) and ΔT_gel(*i-*0)_s in solutions of 3 M monosaccharide unit concentrations (3 M 6-C and 1.5 M 12-C sweetener solutions) (abbreviated ΔT_gel(3M-0)_ in table) of a starch in respect to the following sweetener solution properties: the number of OH groups for intermolecular H-bonding (N*_OH,s_*) on the sweetener, calculated average number of equatorial and exocyclic hydroxyl groups on the sweetener in solution, dry glass transition temperature of the sweetener, sweetener solute molar volume, and sweetener capacity factor (*K*_c_).

	Correlation Coefficients (R)									
	N*_OH,s_*		Equatorial and Exocyclic OHs		Dry T_g_ (°C)		Molar Volume (cm^3^/mol)		Capacity Factor (*K_c_*)	
Starch	log T_gel_s	ΔT_gel(3M-0)_	log T_gel_s	ΔT_gel(3M-0)_	log T_gel_s	ΔT_gel(3M-0)_	log T_gel_s	ΔT_gel(3M-0)_	log T_gel_s	ΔT_gel(3M-0)_
Waxy	0.087	0.260	0.747 **	0.771 **	0.296	0.506	0.589 *	0.767 **	0.385	0.099
Dent corn	0.188	0.342	0.755 **	0.780 **	0.379	0.563 *	0.703 **	0.812 **	0.084	0.389
HACS55	0.094	0.294	0.558 *	0.649 **	0.321	0.543	0.444	0.605 *	0.409	0.087
HACS70	0.075	0.145	0.662 **	0.743 **	0.306	0.358	0.599 *	0.618 *	0.522	0.310
Potato	0.361	0.039	0.024	0.159	0.164	0.197	0.340	0.077	0.066	0.321
Wheat	0.074	0.204	0.606 **	0.676 **	0.315	0.419	0.623 *	0.770 **	0.272	0.122

* *p* values were < 0.10; ** *p*-values were < 0.05

**Table 5:** Comparisons of correlation coefficients (R) of slopes of log T_gel_ vs. sweetener concentration (abbreviated log T_gel_ in table) and ΔT_gel(*i-*0)_s in solutions of 3 M monosaccharide unit concentrations (3 M 6-C and 1.5 M 12-C sweetener solutions) (abbreviated ΔT_gel(3M-0)_ in table) of a sweetener in respect to average amylopectin chain length, percentage of DP 6–12 chains, percentage of DP 13–24 chains, percentage of DP 25–36, and percentage of DP ≥ 37 chains.

	Correlation Coefficients (R)									
	Average Chain Length		% DP 6–12		% DP 13–24		% DP 25–36		% DP ≥ 37	
Sugar	log T_gel_	ΔT_gel(3M-0)_	log T_gel_	ΔT_gel(3M-0)_	log T_gel_	ΔT_gel(3M-0)_	log T_gel_	ΔT_gel(3M-0)_	log T_gel_	ΔT_gel(3M-0)_
Fructose	−0.145	0.261	0.35	−0.043	−0.049	−0.143	−0.764 *	−0.527	−0.078	0.341
Galactose	−0.734 *	NA	0.747 *	NA	−0.174	NA	−0.633	NA	−0.813 *	NA
Glucose	−0.454	−0.385	0.649	0.547	0.048	0.107	−0.900 **	−0.751 *	−0.394	−0.321
Isomalt	−0.774 *	−0.773 *	0.914 **	0.820 **	0.365	0.551	−0.933 **	−0.633	−0.702	−0.708
Isomaltulose	−0.746 *	−0.778 *	0.842 **	0.808 *	0.007	−0.016	−0.741 *	−0.541	−0.748 *	−0.810 **
Maltitol	−0.857 **	−0.886 **	0.938 **	0.904 **	0.220	0.311	−0.761 *	−0.589	−0.838 **	−0.877 **
Maltose	−0.758 *	−0.677	0.881 **	0.674	0.266	0.114	−0.820 **	−0.343	−0.707	−0.695
Mannose	−0.025	0.498	0.255	−0.302	0.057	0.066	−0.761 *	−0.318	0.076	0.616
Sorbitol	−0.674	−0.111	0.822 **	0.137	0.075	−0.631	−0.873 **	0.104	−0.640	−0.207
Sucrose	−0.628	−0.649	0.728	0.623	−0.146	−0.110	−0.567	−0.210	−0.646	−0.711
Trehalose	−0.787 *	−0.687	0.894 **	0.718	0.188	0.345	−0.840 *	−0.521	−0.756 *	−0.655

* *p* values were < 0.10; ** *p*-values were < 0.05.

**Table 6:** Comparisons of correlation coefficients (R) of slopes of log T_gel_ vs. sweetener concentration (abbreviated log T_gel_ in table) and ΔT_gel(*i-*0)_s in solutions of 3 M monosaccharide unit concentrations (3 M 6-C and 1.5 M 12-C sweetener solutions) (abbreviated ΔT_gel(3M-0)_ in table) of a sweetener in respect to the number of building blocks per cluster (NBbl), and inter-block chain length (IB-CL).

	Correlation Coefficients (R)			
	NBbl		IB-CL	
Sugar	log T_gel_	ΔT_gel(3M-0)_	log T_gel_	ΔT_gel(3M-0)_
Fructose	−0.451	−0.65	−0.405	−0.026
Galactose	0.717	NA	−0.613	NA
Glucose	−0.116	0.247	−0.65	−0.369
Isomalt	0.302	0.674	−0.856 **	−0.675
Isomaltulose	0.447	0.709	−0.717	−0.619
Maltitol	0.504	0.742 *	−0.863 **	−0.769 *
Maltose	0.509	0.918 **	−0.765 *	−0.434
Mannose	−0.572	−0.715	−0.356	0.207
Sorbitol	0.163	0.651	−0.792 *	0.011
Sucrose	0.587	0.897 **	−0.609	−0.427
Trehalose	0.413	0.786 *	−0.774 *	−0.482

* *p* values were < 0.10; ** *p*-values were < 0.05.

## Supplementary Materials

The following are available online at https://www.mdpi.com/2304-8158/9/6/757/s1, Figure S1: DSC thermograms of starch slurries (1:2 *w/w*) in water and the measured onset temperature, peak temperature, and ∆H of gelatinization, Table S1: The onset T_gel_s of wheat starch in sweetener solutions grouped by similar solution solids content (same monomeric unit concentration) as follows: Group 1 contains 1 M mono-, 0.5 M disaccharide solutions; Group 2 contains 2 M mono-, 1 M disaccharide solutions; Group 3 contains 3 M mono- and 1.5 M disaccharide solutions; and Group 4 contains 4 M mono- and 2 M disaccharide, Table S2: Pearson correlation coefficients (r) and P-values of T_gel_s and sweetener solution viscosities (log(centipoise)). Solution viscosities were from Allan and others (2018), Figure S2: Wheat starch T_gel_ onsets in sweetener solutions in respect to the effective water content (*φ_W_, eff*) reported in van der Sman and Mauer (2019), Table S3: The onset T_gel_s of waxy corn starch in sweetener solutions grouped by similar solution solids content (same monomeric unit concentration) as follows: Group 1 contains 1 M mono-, 0.5 M disaccharide solutions; Group 2 contains 2 M mono-, 1 M disaccharide solutions; Group 3 contains 3 M mono- and 1.5 M disaccharide solutions; and Group 4 contains 4 M mono- and 2 M disaccharide, Figure S3: Waxy corn starch T_gel_ onsets in sweetener solutions in respect to the effective water content (*φ_W_, eff*) reported in van der Sman and Mauer (2019), Table S4: The onset T_gel_s of dent corn starch in sweetener solutions grouped by similar solution solids content (same monomeric unit concentration) as follows: Group 1 contains 1M mono-, 0.5 M disaccharide solutions; Group 2 contains 2 M mono-, 1 M disaccharide solutions; Group 3 contains 3 M mono- and 1.5 M disaccharide solutions; and Group 4 contains 4 M mono- and 2 M disaccharide, Figure S4: Dent corn starch T_gel_ onsets in sweetener solutions in respect to the effective water content (*φ_W_, eff*) reported in van der Sman and Mauer (2019), Table S5: The onset T_gel_s of HACS55 starch in sweetener solutions grouped by similar solution solids content (same monomeric unit concentration) as follows: Group 1 contains 1M mono-, 0.5 M disaccharide solutions; Group 2 contains 2 M mono-, 1 M disaccharide solutions; Group 3 contains 3 M mono- and 1.5 M disaccharide solutions; and Group 4 contains 4 M mono- and 2 M disaccharide, Figure S5: HACS55 T_gel_ onsets in sweetener solutions in respect to the effective water content (*φ_W_, eff*) reported in van der Sman and Mauer (2019), Table S6: The onset T_gel_s of HACS70 starch in sweetener solutions grouped by similar solution solids content (same monomeric unit concentration) as follows: Group 1 contains 1M mono-, 0.5 M disaccharide solutions; Group 2 contains 2 M mono-, 1 M disaccharide solutions; Group 3 contains 3 M mono- and 1.5 M disaccharide solutions; and Group 4 contains 4 M mono- and 2 M disaccharide, Figure S6: HACS70 T_gel_ onsets in sweetener solutions in respect to the effective water content (*φ_W_, eff*) reported in van der Sman and Mauer (2019), Table S7: The onset T_gel_s of potato starch in sweetener solutions grouped by similar solution solids content (same monomeric unit concentration) as follows: Group 1 contains 1 M mono-, 0.5 M disaccharide solutions; Group 2 contains 2 M mono-, 1 M disaccharide solutions; Group 3 contains 3 M mono- and 1.5 M disaccharide solutions; and Group 4 contains 4 M mono- and 2 M disaccharide, Figure S7: Potato starch T_gel_ onsets in sweetener solutions in respect to the effective water content (*φ_W_, eff*) reported in van der Sman and Mauer (2019), Table S8: Comparisons of correlation coefficients (R) of the log T_gel_ slopes and ΔT_gel(*i-*0)_s in 3 M monomeric unit concentrations (ΔT_gel(3M-0)_) of a sweetener in respect to the amylose contents of starches, Table S9: Averages with 1 standard deviation and P value of Log T_gel_s and ΔT_gel(3M-0)_ in A and B-type starches.
