# Supplementary material for: Effects of Sugars and Sugar Alcohols on the Gelatinization Temperatures of Wheat, Potato, and Corn Starches
Source: Foods. 2020 Jun 8;9(6):757. doi: 10.3390/foods9060757 (PMC7353504; doi:10.3390/foods9060757)
Supplement: Supplementary file 1 [file foods-09-00757-s001.pdf]

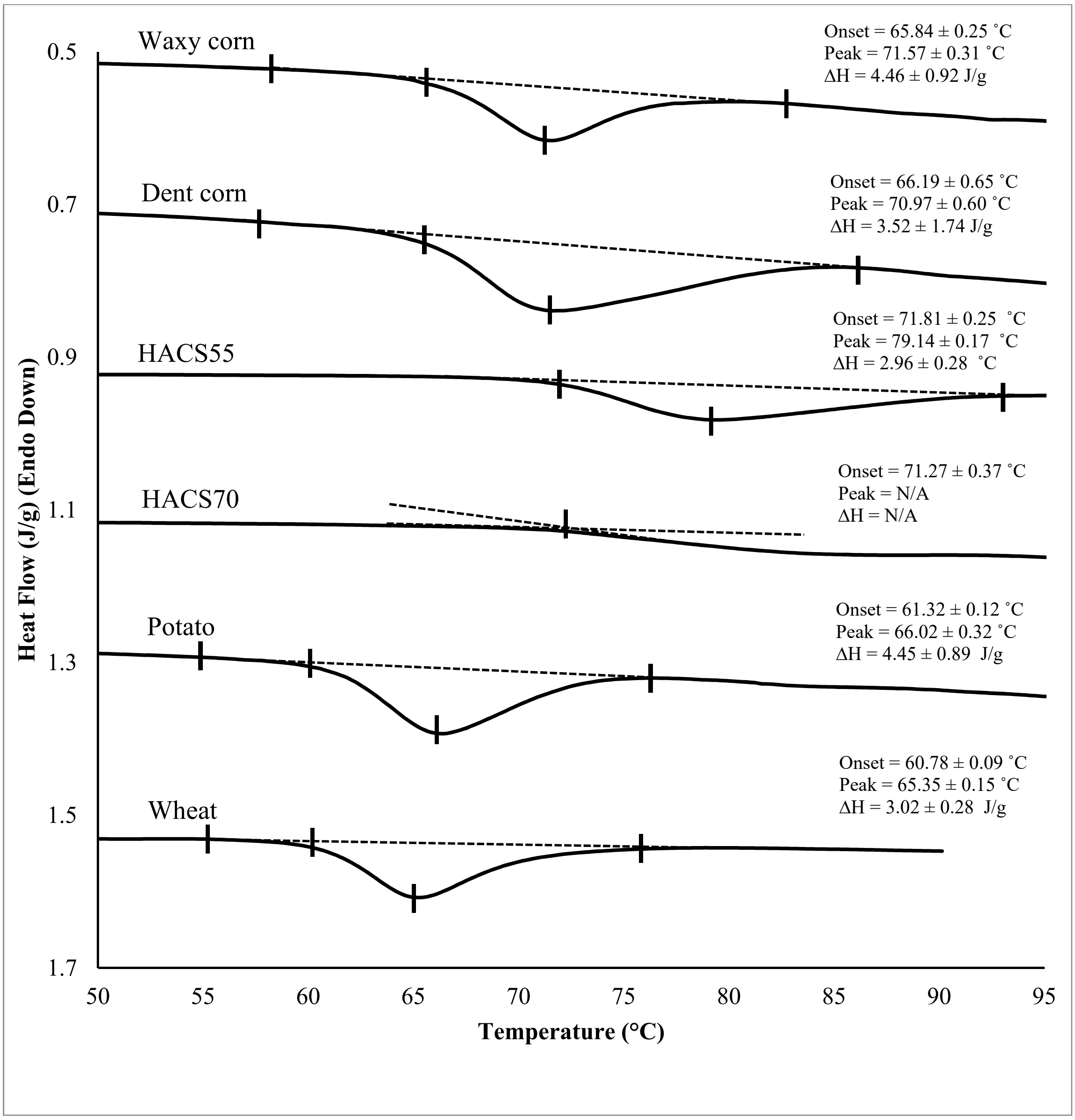

Figure S1. DSC thermograms of starch slurries (1:2 w/w) in water and the measured onset temperature, peak temperature, and ΔH of gelatinization.

Table S1. The onset T_{gel}s of wheat starch in sweetener solutions grouped by similar solution solids content (same monomeric unit concentration) as follows: Group 1 contains 1M mono-, 0.5M disaccharide solutions; Group 2 contains 2M mono-, 1M disaccharide solutions; Group 3 contains 3M mono- and 1.5M disaccharide solutions; and Group 4 contains 4M mono- and 2M disaccharide.

Sweetener	Onset T_{gel} (˚C) Averages, Standard Deviations, and Statistical Groups															
	Group 1*				Group 2				Group 3				Group 4			
Fructose	63.87	±	0.13	BCD	69.04	±	0.10	A	75.12	±	0.19	A	82.22	±	0.10	A
Galactose	64.53	±	0.17	DE	71.13	±	0.42	CD	N/A				N/A			
Mannose	63.91	±	0.12	BCD	68.45	±	0.24	A	74.48	±	0.12	A	82.21	±	0.08	A
Glucose	64.55	±	0.08	DE	70.41	±	0.20	BC	77.18	±	0.51	B	86.19	±	0.64	B
Sorbitol	64.76	±	0.12	E	71.62	±	0.68	DE	78.91	±	0.38	C	88.76	±	0.03	C
Sucrose	64.32	±	0.12	CDE	72.28	±	0.16	EF	79.98	±	0.07	D	89.64	±	0.13	D
Isomaltulose	63.67	±	0.33	BC	73.16	±	0.30	F	83.45	±	0.49	E	N/A			
Isomalt	64.26	±	0.47	CDE	73.22	±	0.54	F	82.33	±	0.49	D	N/A			
Trehalose	64.51	±	0.24	DE	71.36	±	0.17	CDE	79.73	±	0.17	CD	N/A			
Maltose	63.10	±	0.61	B	70.10	±	0.13	B	77.39	±	0.37	B	N/A			
Maltitol	63.83	±	0.19	BCD	72.05	±	0.22	DE	81.36	±	0.23	D	N/A			

Statistical differences are indicated within the concentration groupings by capital letters (vertical columns).
*The control water starch slurry (onset T_{gel} = 60.78 ± 0.09 ˚C) was included in Group 1 statistical analysis and was in statistical group A.

Table S2. Pearson correlation coefficients (r) and P-values of T_{gel}s and sweetener solution viscosities (log(centipoise)). Solution viscosities were from Allan and others (2018).

Starch	r	P-value
Dent Corn	0.98	<0.001
HA55CS	0.99	<0.001
HA70CS	0.96	<0.001
Potato	0.99	<0.001
Waxy Corn	0.98	<0.001
Wheat	0.97	<0.001

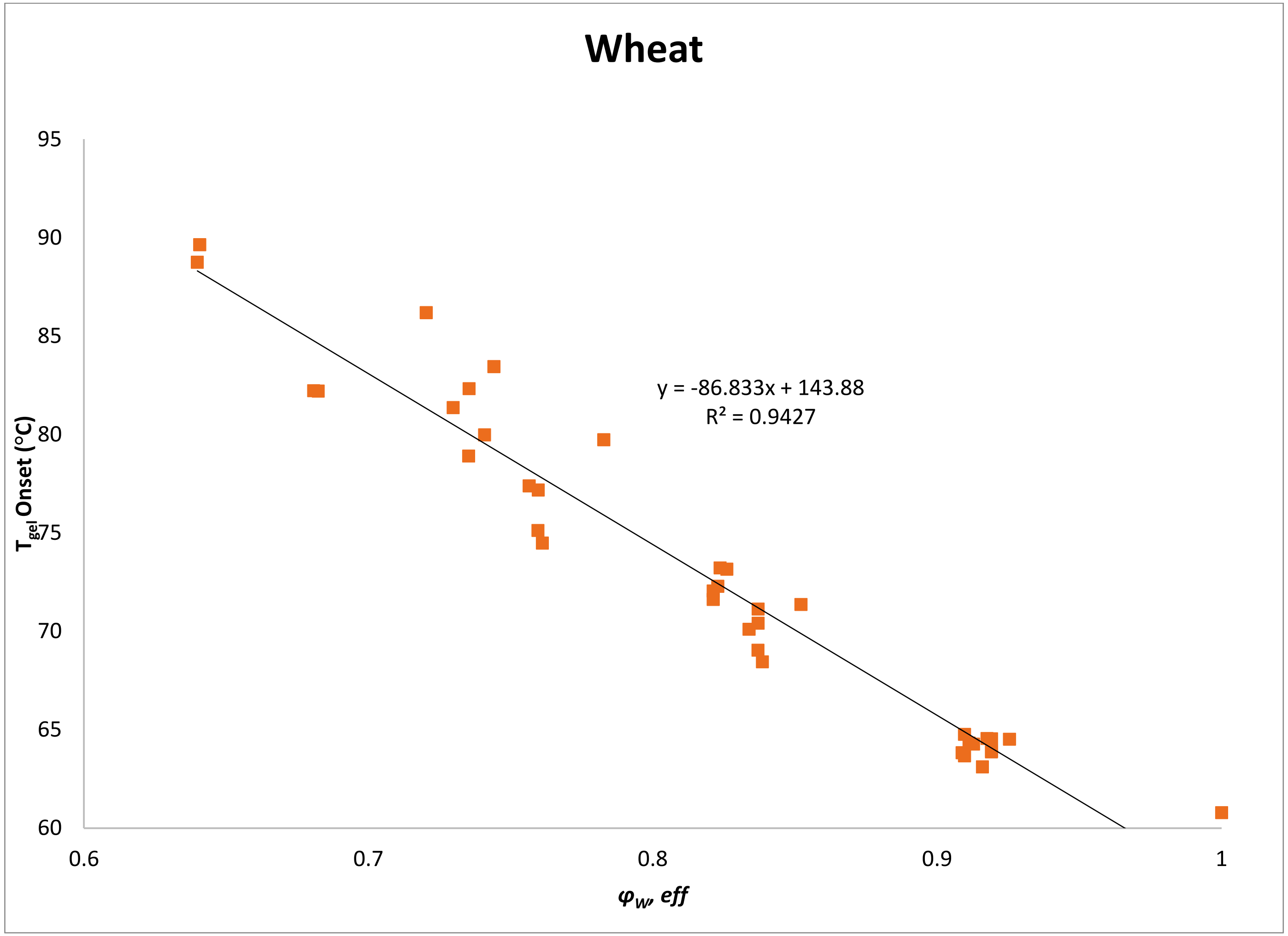

Figure S2. Wheat starch T_{gel} onsets in sweetener solutions in respect to the effective water content (*φw, eff*) reported in van der Sman and Mauer (2019).

Table S3. The onset T_{gel}s of waxy corn starch in sweetener solutions grouped by similar solution solids content (same monomeric unit concentration) as follows: Group 1 contains 1M mono-, 0.5M disaccharide solutions; Group 2 contains 2M mono-, 1M disaccharide solutions; Group 3 contains 3M mono- and 1.5M disaccharide solutions; and Group 4 contains 4M mono- and 2M disaccharide.

Sweetener	Onset T_{gel} (˚C) Averages and Standard Deviations															
	Group 1*				Group 2				Group 3				Group 4			
Fructose	69.23	±	0.19	B	73.32	±	0.24	A	79.19	±	0.11	A	84.92	±	0.25	A
Galactose	69.71	±	0.32	BCD	74.57	±	0.78	BC		N/A				N/A		
Mannose	69.68	±	0.16	BC	74.00	±	0.40	AB	80.09	±	0.41	AB	87.34	±	0.44	B
Glucose	70.03	±	0.19	CDE	75.62	±	0.11	D	80.91	±	0.34	B	89.26	±	1.05	B
Sorbitol	69.90	±	0.21	BCD	75.83	±	0.21	D	81.39	±	0.13	BC	93.62	±	0.37	C
Sucrose	70.40	±	0.26	DEF	75.93	±	0.21	D	82.64	±	0.54	CD	92.25	±	1.43	C
Isomaltulose	70.62	±	0.17	EF	75.75	±	0.05	D	84.32	±	1.08	E		N/A		
Isomalt	70.83	±	0.15	F	77.83	±	0.17	E	86.98	±	0.97	F		N/A		
Trehalose	69.91	±	0.33	BCD	75.52	±	0.10	D	83.66	±	0.24	DE		N/A		
Maltose	70.70	±	0.12	EF	75.54	±	0.40	CD	80.44	±	0.59	AB		N/A		
Maltitol	70.05	±	0.38	CDE	76.31	±	0.21	D	84.87	±	0.47	E		N/A		

Statistical differences are indicated within the concentration groupings by capital letters (vertical columns).

*The control water starch slurry (onset T_{gel} = 65.84± 0.25 ˚C) was included in Group 1 statistical analysis and was in statistical group A.

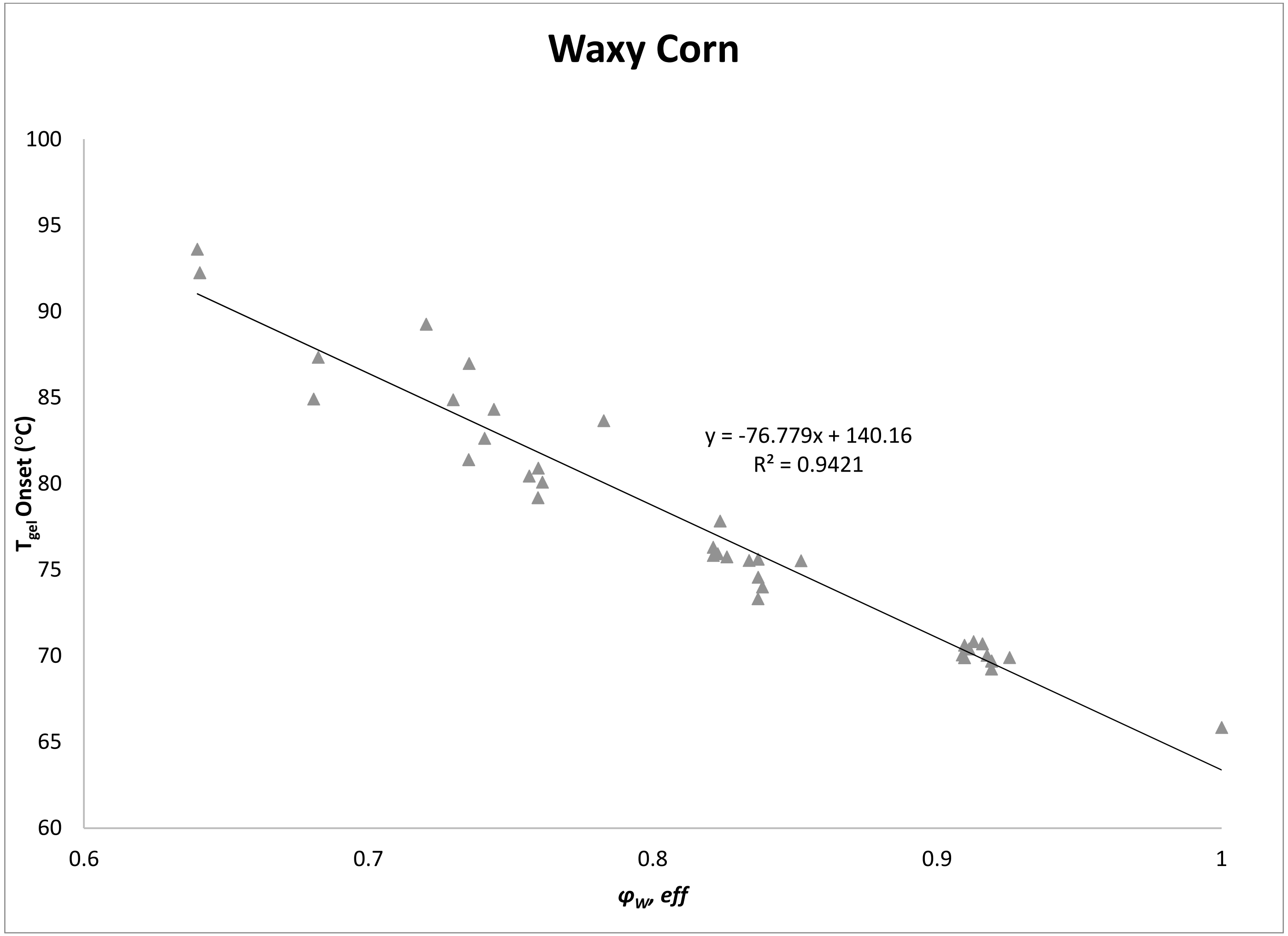

Figure S3. Waxy corn starch T_{gel} onsets in sweetener solutions in respect to the effective water content (*φW, eff*) reported in van der Sman and Mauer (2019).

Table S4. The onset T_{gel}s of dent corn starch in sweetener solutions grouped by similar solution solids content (same monomeric unit concentration) as follows: Group 1 contains 1M mono-, 0.5M disaccharide solutions; Group 2 contains 2M mono-, 1M disaccharide solutions; Group 3 contains 3M mono- and 1.5M disaccharide solutions; and Group 4 contains 4M mono- and 2M disaccharide.

Sweetener	Onset T_{gel} (˚C) Averages, Standard Deviations, and Statistical Groups															
	Group 1*				Group 2				Group 3				Group 4			
Fructose	69.96	±	0.07	B	73.59	±	0.19	A	80.82	±	0.28	A	87.38	±	0.56	A
Galactose	70.27	±	0.12	BC	75.01	±	0.39	B		N/A				N/A		
Mannose	70.45	±	0.35	BC	74.91	±	0.30	B	81.69	±	0.50	A	88.96	±	0.47	B
Glucose	70.38	±	0.57	BC	76.72	±	0.43	CD	83.09	±	0.46	B	91.36	±	0.70	C
Sorbitol	70.97	±	0.13	CD	76.99	±	0.12	CD	83.06	±	0.18	B	95.02	±	0.49	D
Sucrose	71.07	±	0.17	CD	77.31	±	0.34	CD	84.33	±	0.36	C	95.87	±	0.12	D
Isomaltulose	71.65	±	0.08	D	77.78	±	0.71	D	86.88	±	0.12	D		N/A		
Isomalt	71.95	±	0.40	D	79.18	±	0.70	E	89.36	±	0.28	E		N/A		
Trehalose	70.59	±	0.45	BC	76.36	±	0.27	C	86.10	±	0.32	D		N/A		
Maltose	70.99	±	0.23	CD	77.06	±	0.23	CD	83.00	±	0.45	B		N/A		
Maltitol	71.17	±	0.14	CD	77.13	±	0.50	CD	86.56	±	0.46	D		N/A		

Statistical differences are indicated within the concentration groupings by capital letters (vertical columns).

*The control water starch slurry (onset T_{gel} = 66.19 ± 0.65 ˚C) was included in Group 1 statistical analysis and was in statistical group A.

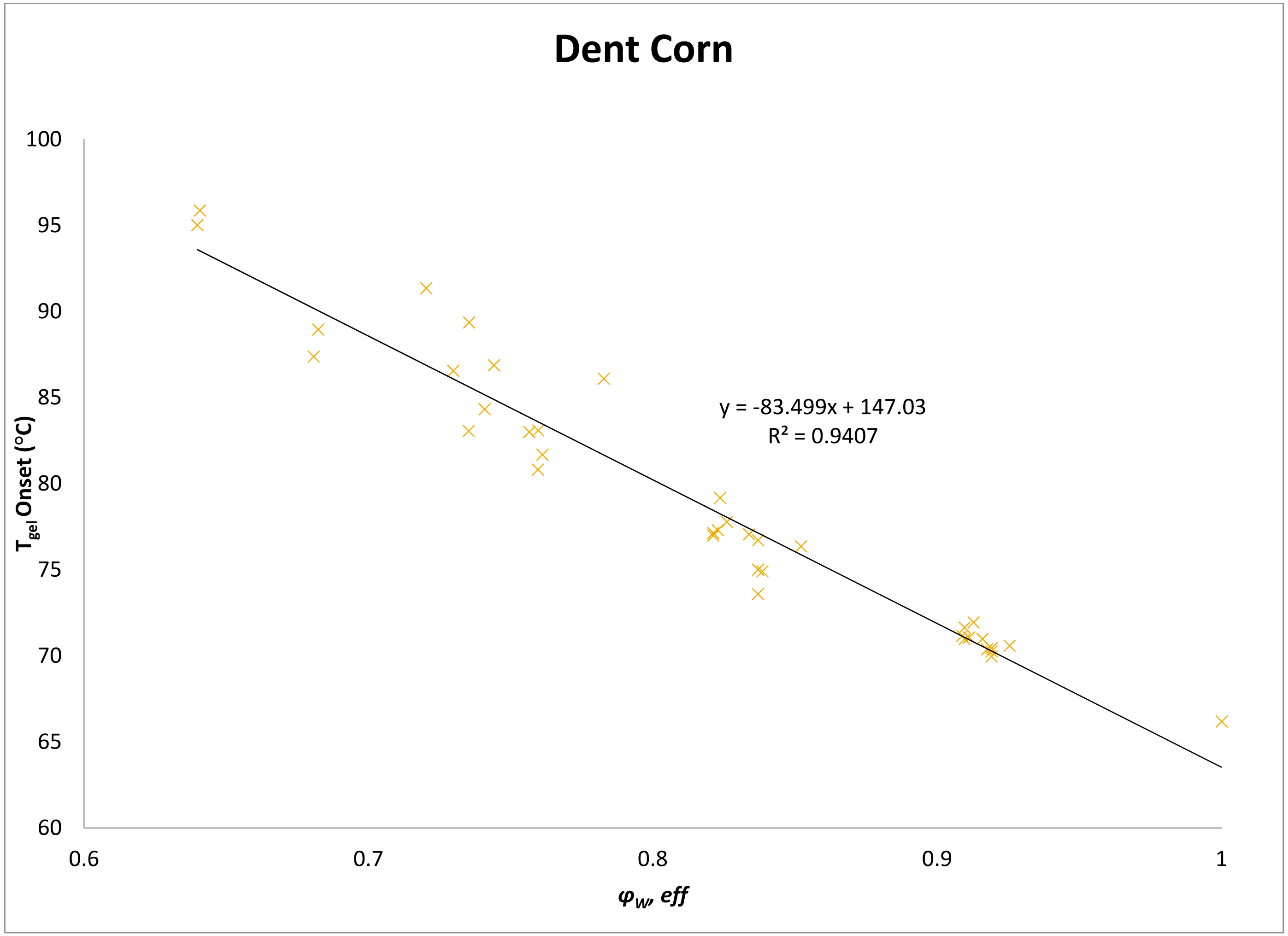

Figure S4. Dent corn starch T_{gel} onsets in sweetener solutions in respect to the effective water content ($\varphi_{W,}$ *eff*) reported in van der Sman and Mauer (2019).

Table S5. The onset T_{gel}s of HACS55 starch in sweetener solutions grouped by similar solution solids content (same monomeric unit concentration) as follows: Group 1 contains 1M mono-, 0.5M disaccharide solutions; Group 2 contains 2M mono-, 1M disaccharide solutions; Group 3 contains 3M mono- and 1.5M disaccharide solutions; and Group 4 contains 4M mono- and 2M disaccharide.

Sweetener	Onset T_{gel} (˚C) Averages, Standard Deviations, and Statistical Groups											
	Group 1*			Group 2			Group 3			Group 4		
Fructose	75.90 ±	0.29	B	79.74 ±	0.59	A	86.20 ±	0.21	A	92.35 ±	0.44	A
Galactose	75.87 ±	0.32	B	80.96 ±	0.25	B	N/A			N/A		
Mannose	76.54 ±	0.11	BC	80.90 ±	0.13	B	86.96 ±	0.11	AB	93.97 ±	0.32	B
Glucose	76.38 ±	0.30	BC	81.97 ±	0.34	CD	87.54 ±	0.29	BC	95.39 ±	0.24	C
Sorbitol	76.24 ±	0.25	BC	82.54 ±	0.12	D	87.71 ±	0.34	BC	98.03 ±	0.01	D
Sucrose	76.15 ±	0.18	BC	81.28 ±	0.18	BC	88.09 ±	0.37	CD	99.50 ±	0.29	E
Isomaltulose	76.79 ±	0.19	C	82.67 ±	0.30	D	89.72 ±	0.60	E	N/A		
Isomalt	76.88 ±	0.33	C	83.71 ±	0.22	E	91.63 ±	0.25	F	N/A		
Trehalose	76.19 ±	0.20	BC	81.81 ±	0.16	BCD	89.66 ±	0.66	E	N/A		
Maltose	76.58 ±	0.28	BC	81.40 ±	0.26	BC	86.30 ±	0.03	A	N/A		
Maltitol	75.92 ±	0.24	B	81.21 ±	0.50	BC	88.95 ±	0.29	DE	N/A		

Statistical differences are indicated within the concentration groupings by capital letters (vertical columns).

*The control water starch slurry (onset T_{gel} = 71.81 ± 0.25 ˚C) was included in Group 1 statistical analysis and was in statistical group A.

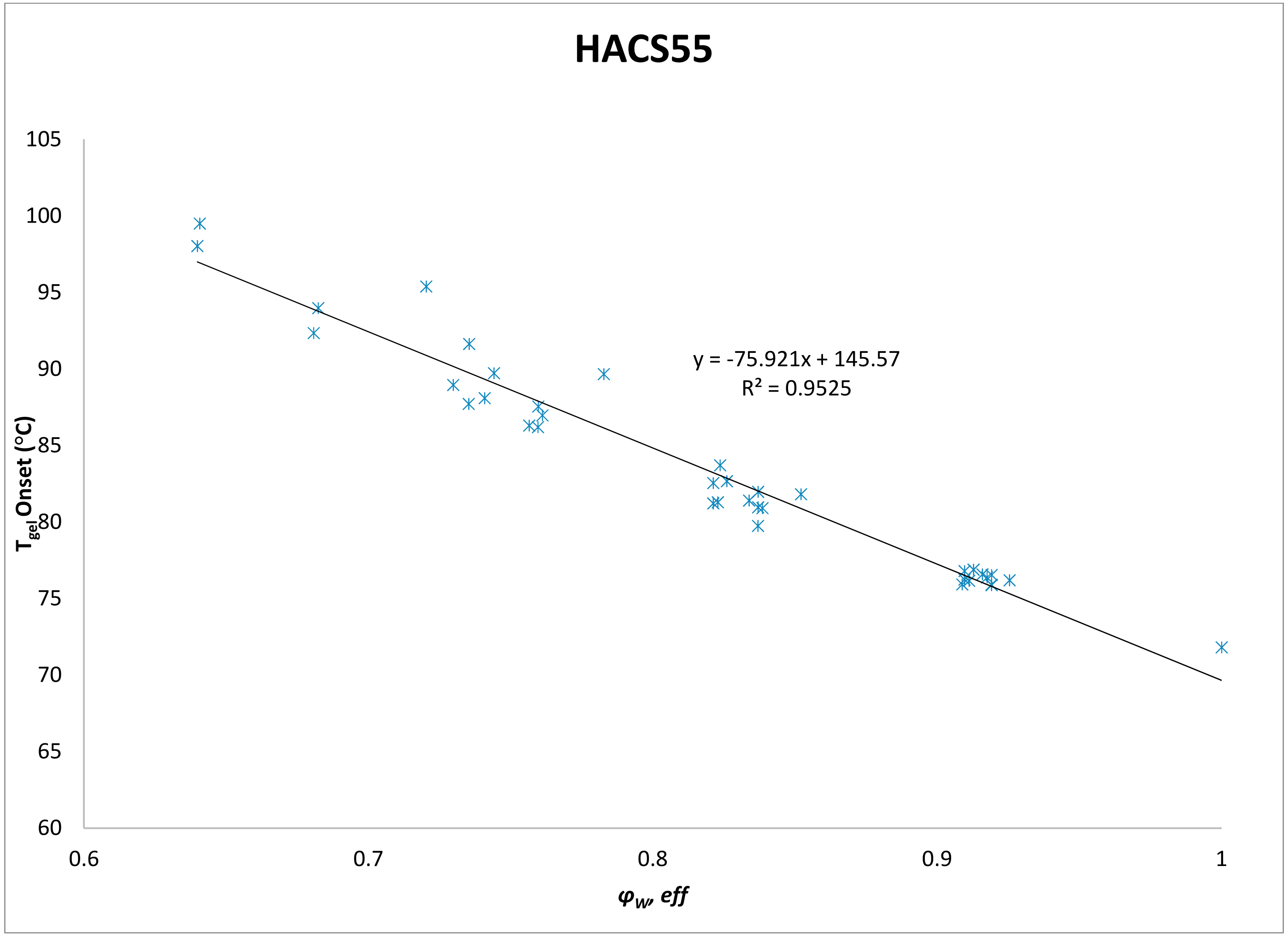

Figure S5. HACS55 T_{gel} onsets in sweetener solutions in respect to the effective water content (*φw, eff*) reported in van der Sman and Mauer (2019).

Table S6. The onset T_{gel}s of HACS70 starch in sweetener solutions grouped by similar solution solids content (same monomeric unit concentration) as follows: Group 1 contains 1M mono-, 0.5M disaccharide solutions; Group 2 contains 2M mono-, 1M disaccharide solutions; Group 3 contains 3M mono- and 1.5M disaccharide solutions; and Group 4 contains 4M mono- and 2M disaccharide.

Sweetener	Onset T_{gel} (˚C) Averages, Standard Deviations, and Statistical Groups															
	Group 1*				Group 2				Group 3				Group 4			
Fructose	75.01	±	0.18	BC	78.40	±	0.31	A	84.61	±	0.49	A	88.36	±	0.80	A
Galactose	75.25	±	0.19	BCD	79.97	±	0.44	B	N/A				N/A			
Mannose	75.00	±	0.19	BC	79.71	±	0.36	AB	85.73	±	0.52	A	92.26	±	0.49	B
Glucose	74.75	±	0.79	B	80.51	±	0.13	BC	86.18	±	0.52	A	94.31	±	1.28	C
Sorbitol	75.49	±	0.23	BCD	80.98	±	0.21	BC	89.02	±	1.21	B	96.65	±	0.22	D
Sucrose	75.33	±	0.30	BCD	80.71	±	0.40	BC	88.63	±	1.48	B	102.98	±	0.19	E
Isomaltulose	75.61	±	0.80	BCD	81.71	±	0.39	CD	89.09	±	0.32	BC	N/A			
Isomalt	76.33	±	0.37	D	82.77	±	0.53	D	91.19	±	0.26	C	N/A			
Trehalose	75.12	±	0.38	BC	80.58	±	0.25	BC	88.71	±	0.33	B	N/A			
Maltose	76.03	±	0.31	CD	81.11	±	0.10	BC	86.07	±	0.59	A	N/A			
Maltitol	75.35	±	0.06	BCD	81.88	±	1.29	CD	89.03	±	0.22	B	N/A			

Statistical differences are indicated within the concentration groupings by capital letters (vertical columns).
*The control water starch slurry (onset T_{gel} = 71.27 ± 0.37 ˚C) was included in Group 1 statistical analysis and was in statistical group A.

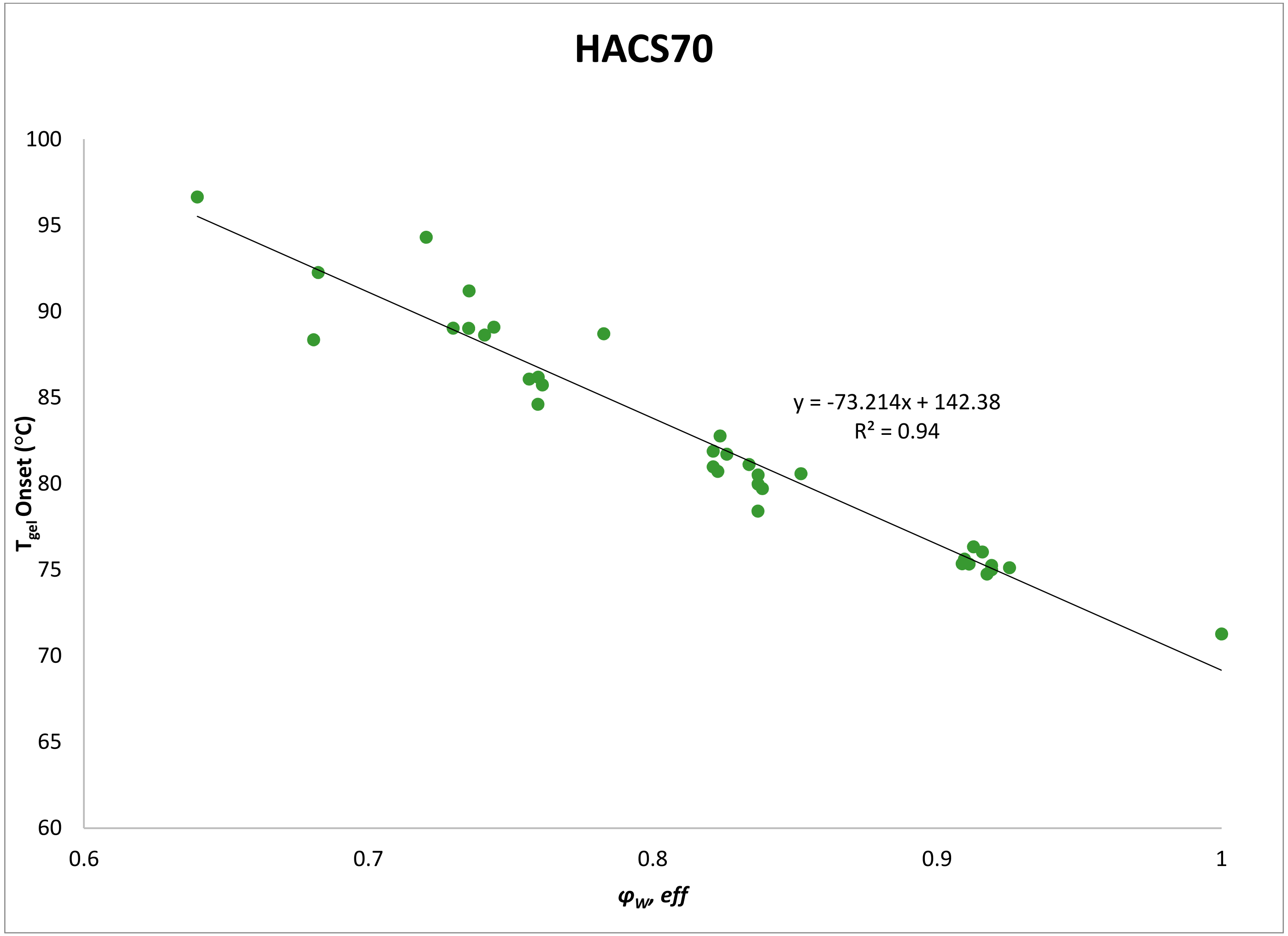

Figure S6. HACS70 T_{gel} onsets in sweetener solutions in respect to the effective water content (*φw, eff*) reported in van der Sman and Mauer (2019).

Table S7. The onset T_{gel}s of potato starch in sweetener solutions grouped by similar solution solids content (same monomeric unit concentration) as follows: Group 1 contains 1M mono-, 0.5M disaccharide solutions; Group 2 contains 2M mono-, 1M disaccharide solutions; Group 3 contains 3M mono- and 1.5M disaccharide solutions; and Group 4 contains 4M mono- and 2M disaccharide.

Sweetener	Onset T_{gel} (˚C) Averages, Standard Deviations, and Statistical Groups															
	Group 1*				Group 2				Group 3				Group 4			
Fructose	65.77	±	0.02	DE	71.03	±	0.55	AB	78.43	±	0.50	CDE	85.92	±	0.03	A
Galactose	65.62	±	0.19	DE	70.88	±	0.07	AB	N/A				N/A			
Mannose	65.71	±	0.25	DE	70.95	±	0.47	AB	78.25	±	0.32	CDE	86.46	±	0.15	B
Glucose	65.51	±	0.33	CDE	71.22	±	0.44	AB	77.51	±	0.63	BCD	87.51	±	0.28	C
Sorbitol	65.20	±	0.55	BCD	71.31	±	0.31	B	77.25	±	0.26	BC	88.52	±	0.12	D
Sucrose	65.17	±	0.01	BCD	70.47	±	0.35	AB	76.90	±	0.64	B	87.11	±	0.13	C
Isomaltulose	65.54	±	0.19	DE	71.31	±	0.29	B	79.19	±	0.29	E	N/A			
Isomalt	65.98	±	0.17	E	72.84	±	0.71	C	81.07	±	0.26	F	N/A			
Trehalose	64.76	±	0.24	B	70.40	±	0.44	AB	78.51	±	0.14	CDE	N/A			
Maltose	65.71	±	0.32	DE	70.60	±	0.17	AB	74.83	±	0.18	A	N/A			
Maltitol	64.80	±	0.23	BC	70.09	±	0.22	A	78.57	±	0.72	DE	N/A			

Statistical differences are indicated within the concentration groupings by capital letters (vertical columns).
*The control water starch slurry (onset T_{gel} = 61.32 ± 0.19 ˚C) was included in Group 1 statistical analysis and was in statistical group A.

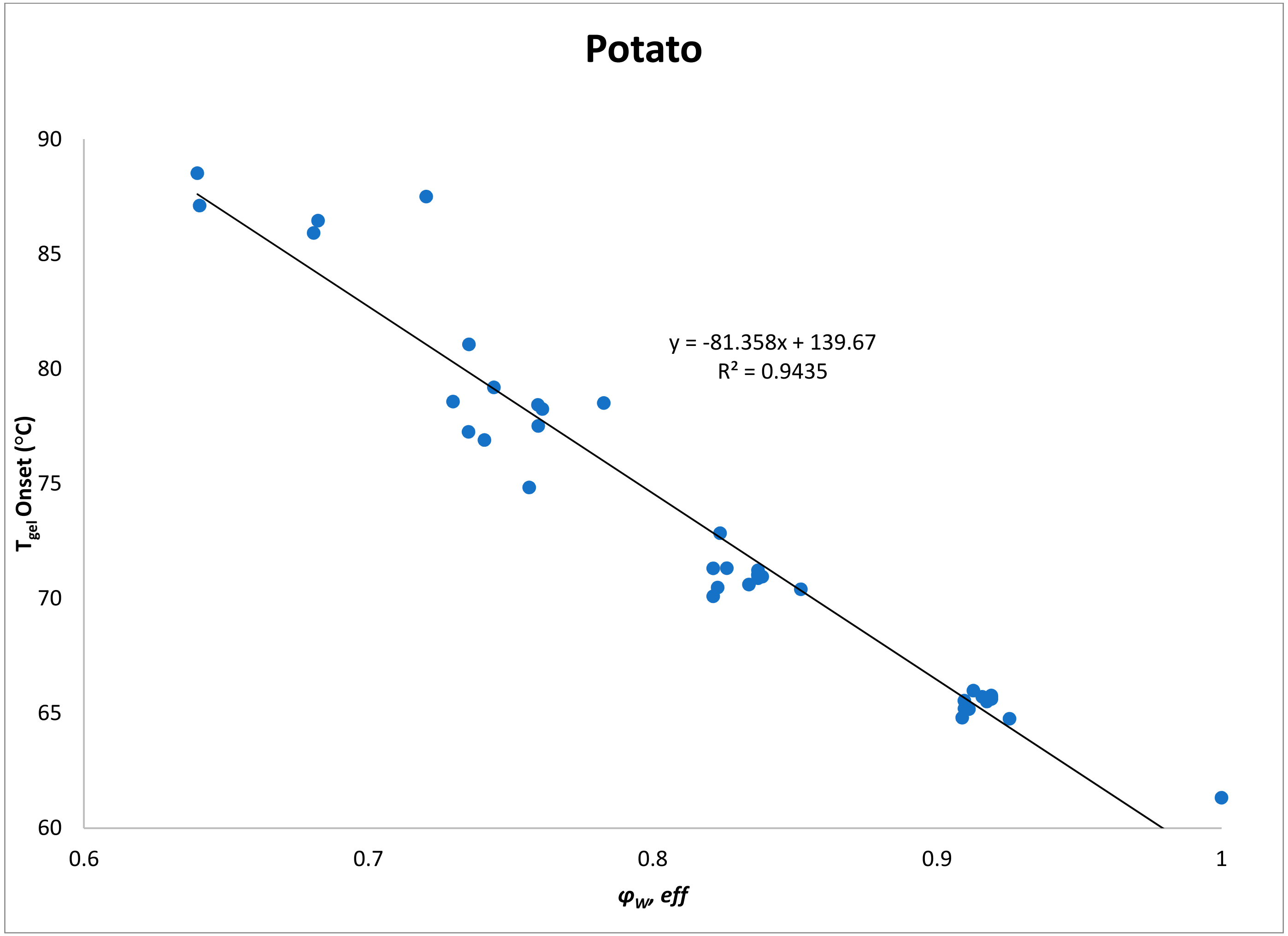

Figure S7. Potato starch T_{gel} onsets in sweetener solutions in respect to the effective water content (*φw, eff*) reported in van der Sman and Mauer (2019).

Table S8. Comparisons of correlation coefficients (R) of the log T_{gel} slopes and $\Delta T_{gel(i\text{-}0)}$s in 3 M monomeric unit concentrations ($\Delta T_{gel(3M\text{-}0)}$) of a sweetener in respect to the amylose contents of starches.

	Pearson Coefficient					
	**Apparent Amylose**		**Absolute Amylose**		**% Crystallinity**	
**Sweetener**	log T_{gel}	$\Delta T_{gel(3M\text{-}0)}$	log T_{gel}	$\Delta T_{gel(3M\text{-}0)}$	log T_{gel}	$\Delta T_{gel(3M\text{-}0)}$
Fructose	-0.291	0.018	-0.297	-0.111	0.566	0.517
Galactose	-0.354	NA	-0.082	NA	-0.067	NA
Glucose	-0.436	-0.162	-0.305	0.007	0.470	0.055
Isomalt	-0.621	-0.494	-0.374	-0.216	0.349	0.019
Isomaltulose	-0.421	-0.318	-0.119	0.034	0.030	-0.242
Maltitol	-0.579	-0.515	-0.262	-0.169	0.143	-0.074
Maltose	-0.470	-0.156	-0.169	0.197	0.125	-0.436
Mannose	-0.274	0.138	-0.337	-0.093	0.693	0.531
Sorbitol	-0.522	0.411	-0.290	0.698	0.330	-0.619
Sucrose	-0.214	-0.105	0.129	0.266	-0.124	-0.489
Trehalose	-0.519	-0.276	-0.237	0.022	0.139	-0.247

* P values were <0.10

** P-values were <0.05

Table S9. Averages with 1 standard deviation and P value of Log T_{gel}s and $\Delta T_{gel(3M\text{-}0)}$ in A and B-type starches.

	Log T_{gel}		$\Delta T_{gel(3M\text{-}0)}$	
**Starch Type**	**Average**	**P-value**	**Average**	**P-value**
A	0.034±0.004	0.004	17.7±2.7	0.063
B	0.031±0.004		16.6±1.7	
